# Genomic and transcriptional alterations in first-line chemotherapy exert a potentially unfavorable influence on subsequent immunotherapy in NSCLC

**DOI:** 10.7150/thno.58039

**Published:** 2021-05-13

**Authors:** Yayi He, Linsong Chen, Lishu Zhao, Shiying Dang, Guifeng Liu, Shinji Sasada, Patrick C. Ma, Nico van Zandwijk, Rafael Rosell, Helmut H. Popper, Hao Wang, Minlin Jiang, Haoyue Guo, Xinyi Liu, Shifu Chen, Xiaoni Zhang, Mingyan Xu, Bo Zhu, Ming Liu, Caicun Zhou

**Affiliations:** 1 Department of Medical Oncology, Shanghai Pulmonary Hospital & Thoracic Cancer Institute, Tongji University School of Medicine, No. 507, Zhengmin Road, Yangpu District, Shanghai 200433, China.; 2 Department of Surgery, Shanghai Pulmonary Hospital & Thoracic Cancer Institute, Tongji University School of Medicine, No. 507, Zhengmin Road, Yangpu District, Shanghai 200433, China.; 3 HaploX Biotechnology, Co., Ltd. 8th floor, Auto Electric Power Building, Songpingshan Road, Nanshan District, Shenzhen 518057, Guangdong, China.; 4 Department of Respiratory Medicine, Tokyo Saiseikai Central Hospital, Minato-ku, Tokyo, Japan.; 5 Penn State Cancer Institute, Penn State Health Milton S. Hershey Medical Center, Pennsylvania State University, Hershey, PA, USA.; 6 Sydney Local Health District, Concord Repatriation General Hospital, Concord, NSW, The University of Sydney, Australia.; 7 Cancer Biology and Precision Medicine Program, Catalan Institute of Oncology, Hospital Germans Trias i Pujol, Ctra. Canyet, Badalona (Barcelona), Spain.; 8 Department of Pathology, Medical University of Graz, Neue Stiftingtalstrasse 6, Graz 8036, Austria.; 9 Institute of Cancer, Xinqiao Hospital, the Army Medical University, No. 83, Xinqiaozheng Street, Shapingba District, Chongqing 400037, China.

**Keywords:** Lung cancer, chemotherapy, immunotherapy, tumor mutational burden (TMB), programmed death-1 (PD-1), messager ribonucleic acid (mRNA).

## Abstract

**Background:** Recent studies in non-small cell lung cancer (NSCLC) patients have demonstrated that first-line immunotherapy is associated with better therapeutic response than second-line treatment. So far, the mechanisms need to be explored. It prompted us to evaluate the association between first-line chemotherapy and subsequent immunotherapy in NSCLC as well as its underlying mechanisms at the genomic and transcriptomic level.

**Methods:** We launched a prospective, observational clinical study, paired tumor biopsies before and after chemotherapy were collected from NSCLC patients without tyrosine kinase inhibitor (TKI)-related driver gene mutations. The analyses included genomic and transcriptional changes performed by next-generation sequencing (NGS)-based whole-exome sequencing (WES) and messager ribonucleic acid (mRNA) sequencing. Characteristic mutational alterations in 1574 genes were investigated based on mutational status, clinicopathological factors, and chemotherapy responses. Gene Ontology (GO) and Kyoto Encyclopedia of Genes and Genomes (KEGG) pathway enrichment analysis, neoantigen prediction and intratumoral heterogeneity evaluation were also performed.

**Results:** Samples and information from 32 NSCLC patients without TKI-related driver gene mutations were obtained. We found that the total number of single nucleotide variants (SNV)/insertion-deletion (INDEL) mutations did not change significantly after chemotherapy. The tumor mutation burden (TMB) decreased significantly after chemotherapy in smoking patients and the decreased TMB correlated with a better survival of smoking patients. The change in copy number variations (CNVs) exhibited a decreasing trend during chemotherapy. Subsequent analysis at mRNA level revealed a significant decrease in the expression levels of genes related to antigen processing and presentation as well as other factors relevant for response to immunotherapy. Pathway enrichment analysis confirmed that the immune-related signaling pathways or biological processes were decreased after first-line chemotherapy.

**Conclusions:** Our study presents an explanation for the unsatisfactory results of immunotherapy when given after chemotherapy, and suggests that first-line chemotherapy is able to influence the tumor microenvironment and decrease the efficacy of subsequent immunotherapy. The study was registered at ClinicalTrials.gov, number NCT03764917, and has completed enrolment; patients are still in follow-up.

## Introduction

Lung cancer remained the top reason for cancer related deaths, and 85% of lung cancers are non-small cell lung cancer (NSCLC) 1. Driver gene mutations can be found in a significant percentage of patients with advanced or metastatic NSCLC 2-5. Patients with mutations of epidermal growth factor receptor (*EGFR*) and anaplastic lymphoma kinase (*ALK*) respond well to the corresponding tyrosine kinase inhibitors (TKIs) 6. However, in a substantial percentage of NSCLC patient, TKI-related driver gene mutation(s) are absent 7-9. For patients with advanced/metastatic NSCLC without a TKI-related driver mutation, chemotherapy remains an important treatment option 10, 11.

Immune checkpoint inhibitors (ICIs) have significantly changed the therapeutic landscape for lung cancer patients 12, 13. At present, platinum-based chemotherapy, rather than ICIs, is still used as the first-line treatment for most advanced NSCLC patients. Considering ICIs require pre-existing immune systems especially T cells to perform anti-neoplastic effects, a hypothesis was proposed that a weak systemic immunity induced by chemotherapy might impede the effect of ICIs. Furthermore, a series of clinical trials have validated this hypothesis 14-16. The KEYNOTE-042 study clearly demonstrated that first-line pembrolizumab is superior to chemotherapy in advanced/metastatic NSCLC patients without a driver mutation 17. Additional studies have confirmed this observation 18, 19. Moreover, immunotherapy given as first-line treatment was associated with a better response rate than immunotherapy given as a posterior line therapy 20. However, these trials did not reveal the molecular and cellular mechanism, which impaired the efficacy of posterior line ICIs.

With the advance of next generation sequencing, multiple features based on gene and transcription level have been proved a critical function in immunotherapy. Tumor mutational burden (TMB) is defined as the number of somatic mutations per deoxyribonucleic acid (DNA) megabase, which is a promising biomarker for ICI efficacy in various cancers 21. Meanwhile, the tumor copy number variations (CNVs) provide a superior prediction in immunotherapeutic response than conventional biomarkers in gastrointestinal cancers 22. Thus, these molecular features offer a new perspective and an excellent practical way to detect the effects of first-line chemotherapy on tumor immune microenvironment (TIME).

This study aims to explore the potential influence of chemotherapy on the response to subsequent immunotherapy and analyze the effects of the first-line chemotherapy on TMB, CNVs, and immune-related factors. To this end, we collected paired pre- and post-therapeutic needle biopsy samples from 32 NSCLC patients without *EGFR/ALK/ROS/MET/RET* or *BRAF* mutations and performed WES on all samples to obtain the mutation spectrum and TMB. Meanwhile, (full) gene expression profiles were obtained by mRNA sequencing of lung cancer tissues from 11 patients with adequate paired tissues. Our data indicated that both TMB and key immune-related gene expression were decreased after chemotherapy. Additionally, pathways relevant to PD-1 blockade were shown to be downregulated, creating an unfavorable microenvironment for subsequent immunotherapy.

## Methods and materials

### Ethical approval by participating hospitals

Experimental plans and protocols for this study (NO K18-203) were approved by the ethics/licensing committee of the Shanghai Pulmonary Hospital. Written informed consents were obtained from all patients participating in the study. All experiments, methods, procedures, and personnel training were carried out in accordance with relevant guidelines and regulations of the participating hospitals and laboratories.

### Study design, patients, and samples

The study was designed by the investigators (Zhou CC, He YY, and Zhu B). Tumor biopsy and blood samples were collected prospectively. The study included NSCLC patients without *EGFR, ALK, ROS1, MET, RET*, or *BRAF* mutations. All patients were diagnosed with late-stage NSCLC (IIIB-IV) for the first time and were expected to receive first-line chemotherapy due to the lack of TKI-related driver gene mutations. Another thing had to be mentioned was that there were two patients whose clinical stages were defined as stage III according to the positive signal of mediastinal lymph nodes on positron emission tomography-computed tomography (PET-CT) received neoadjuvant chemotherapy and surgery but their pathological stages were corrected as stage I and stage II after surgery respectively because of the negative report of pathological examination of mediastinal lymph nodes. Thus, in the manuscript we chose pathological stage as the final stage of these two patients. These patients had no history of cancer or cancer therapy. In this way, samples from 32 NSCLC patients without driver mutations were obtained, and information on patients' clinical status was collected (Figure S1, S2). Samples included fresh or frozen samples taken from needle biopsy and blood samples obtained at the same time during biopsy. The pre-therapeutic tissue and blood samples, were collected after diagnosis and before therapy, whereas the post-therapeutic samples were collected after 4-12 cycles of chemotherapy (e.g., before the start of the 5^th^ or the 13^th^ cycle). The evaluation of response was according to RECIST 1.1. Briefly, partial response (PR) was defined as the length of target lesion decreased 30% or more than baseline, and progressed disease (PD) was defined as the length of target lesion increased 20% or more than the smallest or a new lesion occurred. Stable disease (SD) was the status between PR and PD. Laboratory technicians were blinded from any of the subjects' clinical information. The NSCLC diagnosis was based on information from imaging and subsequent pathological examination. None of the patients enrolled in this study received chemotherapy, radiotherapy, targeted therapy, or immunotherapy before tissue or blood samples were collected. The somatic sequencing data presented in this study were from lung tumor tissue DNA, and germline sequencing data were from the corresponding white blood cell genomic DNA. All patients in this study received the first-line chemotherapy; the regimens and the responses assessed are presented in Table S1. The study was registered at ClinicalTrials.gov, number NCT03764917, and has completed enrolment; patients are still in follow-up.

### Sample preparation, targeted next-generation sequencing (NGS), and data processing for whole-exome sequencing (WES)

DNA was extracted from the needle biopsy samples using the QIAamp DNA tissue Kit (QIAGEN, Valencia, CA, USA) following the manufacturer's instructions. The whole exome sequencing on tissue samples was performed as previously described 23. For blood samples, 10 ml blood was collected in EDTA tubes and centrifuged at 1,600 × g for 10 min (4 °C) within 2 h of collection. The peripheral blood lymphocyte (PBL) debris was stored at -20 °C for later use. The supernatants were further centrifuged at 10,000 × g for 10 min (4 °C), and plasma was harvested and stored at -80 °C for later use. DNA was extracted from PBLs using the RelaxGene Blood DNA system (Tiangen Biotech Co., Ltd., Beijing, China). Both cancer tissue and white blood cell genomic DNA were quantified with the Qubit 2.0 Fluorometer and the Qubit dsDNA HS assay kit (Thermo Fisher Scientific, Inc., Waltham, MA, USA) according to manufacturer's instructions. In brief, fragmented genomic DNA underwent end-repairing, A-tailing and ligation were sequentially completed with indexed adapters, followed by size selection using Agencourt AMPure XP beads (Beckman Coulter Inc., Brea, CA, USA), and DNA fragments were used for library construction with the KAPA Library Preparation kit (Kapa Biosystems, Inc., Wilmington, MA, USA) according to the manufacturer's protocol. Hybridization-based target enrichment was carried out with HaploX WESPlus gene panel (an upgraded version of the standard WES, HaploX Biotechnology) for cancer tissue sequencing. Seven to eight polymerase chain reaction (PCR) cycles, depending on the amount of DNA input, were performed on Pre‑LM‑PCR Oligos (Kapa Biosystems, Inc.) in 50 μl reactions. DNA sequencing was then performed on the Illumina Novaseq 6000 system according to the manufacturer's instructions at an average depth of 500×.

Genome Analysis Toolkit (GATK, Version 4.1.7.0) best practice workflow was followed for somatic short variant discovery. Sequencing data were aligned to the hg19 genome (GRch37) using Burrows‑Wheeler Aligner (BWA, Version: 0.7.17-r1198) with default settings. Duplicated reads were subsequently marked and removed using the GATK Picard tool. After the base quality score recalibration using BaseRecalibrator and ApplyBQSR functions of GATK, SNVs and INDELs were called from tumor and matched-normal pairs using Mutect2 from GATK. MutSigCV (Version: 1.41) was used to determine significantly mutated gene with a q value below 0.05. Matched genomic DNA from white blood cells was used as control.

The exaction of the mutational signatures in our tumor samples was performed with SignatureAnalyzer. Non-negative matrix factorization algorithm (NMF) was applied for mutational signature analysis. The mutational signatures detected in our samples were compared to 30 known COSMIC cancer signatures. ConsensusClusterPlus was used for classifying patients according to their mutational signature. We selected 80% item resampling (pItem), 10 resamplings (reps), Pearson correlation distances (distance) as settings of the ConsensusClusterPlus.

### Sample preparation, targeted NGS, and data processing for mRNA sequencing

#### RNA-sequencing library preparation

Total RNA was extracted using Trizol (Life Technologies Corp.) and further treated with DNase to remove genomic DNA contamination. Isolation of mRNA was performed using the NEBNext PolyA mRNA Magnetic Isolation Module (New England Biolabs, Ipswich, MA, USA), and the mRNA was then used for RNA-sequencing library preparation with the NEB Next Ultra Directional RNA Library Prep Kit for Illumina (New England Biolabs, Ipswich, MA, USA). The library was then subjected to Illumina sequencing with the paired-end 2 x 150 sequencing mode.

#### Quality control and alignment of sequencing data

Raw reads were applied quality and adapter trimming using Trim Galore (v0.5.0). FastQC (v0.11.8) was used to ensure high read quality 24. The clean reads were mapped to the human genome using the HISAT2 software. After mapping, read counts for each transcript/gene were calculate using featureCounts (v1.6.3).

#### Identified differentially expressed genes (DEGs) and functional annotation

We used “edgeR” R package to calculate the DEGs and FDR less than 0.05 and |log2 (FC)| higher than 2 were set as cutoff values. Gene ontology (GO) and Kyoto Encyclopedia of Genes and Genomes (KEGG) analysis were conducted through DAVID (https://david.ncifcrf.gov/).

#### Transcription factor analysis and protein-protein interaction (PPI) network construction

The transcription factor was conducted on TRRUST (https://www.grnpedia.org/trrust/). The PPI network was constructed through STRING (https://string-db.org/).

### Prediction of Neoantigen

WES data were reviewed for non-synonymous exonic mutations (NSEM), and the binding affinity with patient-restricted major histocompatibility complex Class I (MHC Class I) molecules of all possible 8-mer-11-mer peptides spanning NSEM was evaluated with the NetMHCPan4.0 (http://www.cbs.dtu.dk/services/NetMHCpan/) algorithm based on patient human leukocyte antigen-A (HLA-A), HLA-B, and HLA-C alleles. Candidate peptides were considered HLA binders when IC50<500 nM, and was considered with high affinity binders when IC50<50 nM 25.

### Statistics and data analysis and calculation of somatic TMB

Statistical analysis was performed and figures were plotted with GraphPad Prism 5.0 software (GraphPad Software, Inc., La Jolla, CA, USA), SPSS 22.0 (SPSS lnc., Chicago, IL,USA) and R software (https://www.r-project.org/). Student's t-test or non-parametric test was performed when 2 groups were compared, and analysis of variance (ANOVA) and post hoc tests were performed when 3 or more groups were compared. Paired t test or Wilcoxon sign rank test was used to analyze the paired data. Chi-square test and Fisher's test were performed when rate or percentage was compared for significance. *P*<0.05 was identified as statistically significant. TMB was calculated by dividing the total number of tissue non-synonymous SNV and INDEL variations (allele frequency > 5%) by the full length of the WES panel. In addition, we used MATH score as quantitative measure for intratumoral heterogeneity (ITH), which considered the width of variant allele frequency distribution for calculation.

## Results

### Analysis of mutation spectrum by WES revealed genetic variations following chemotherapy

To investigate the genetic alterations occurring during chemotherapy in NSCLC patients, we obtained paired lung cancer tissues before and after the first-line chemotherapy and compared the genetic alterations at both the genomic and transcriptomic levels using WES and mRNA sequencing. WES was performed using 32 paired tissues and the corresponding normal white blood cells as the control, and mRNA sequencing was performed using 11 paired tissues.

**Figure 1:**
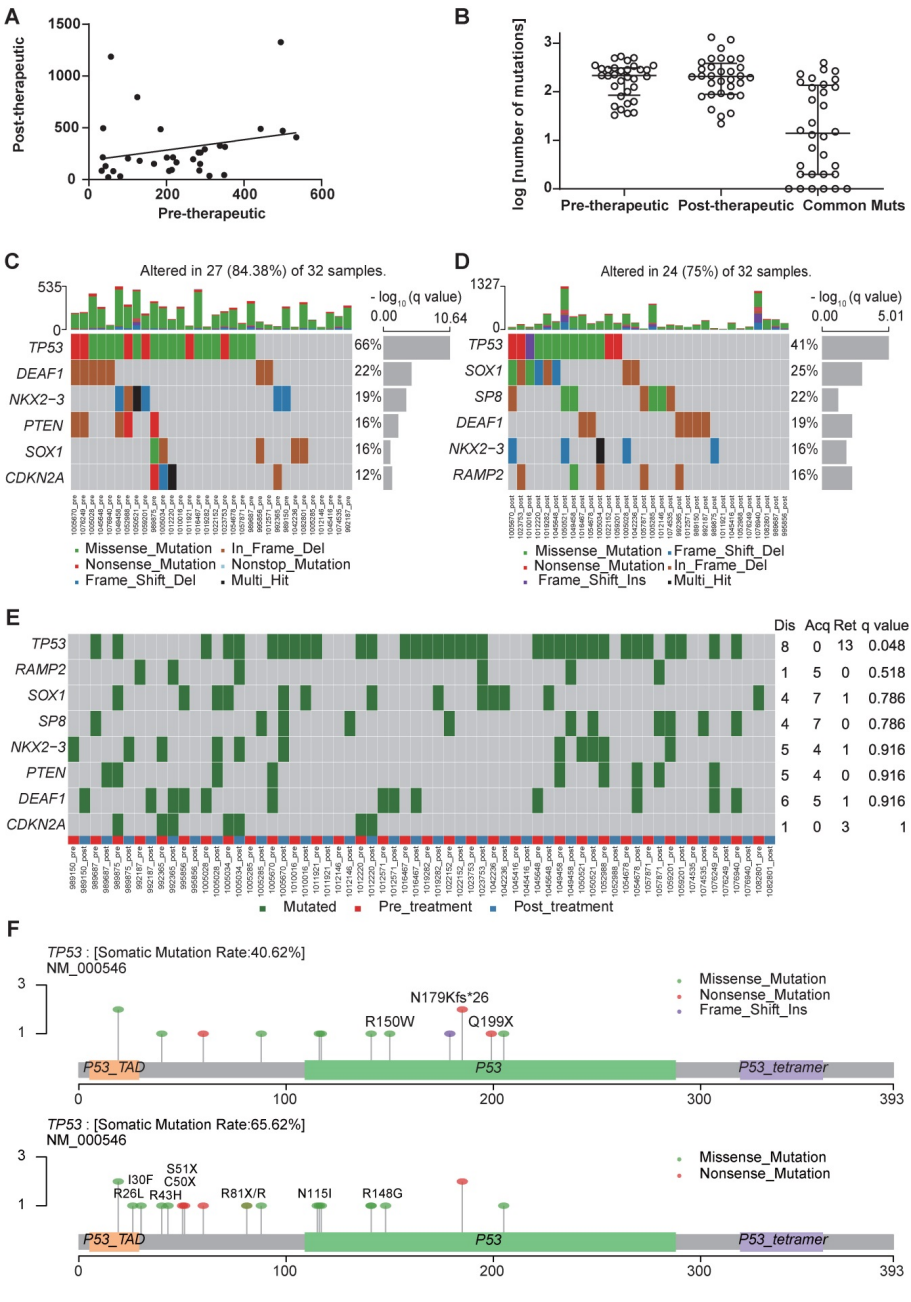
The first-line chemotherapy led to genomic changes in lung cancer tissues. (A) a linear relationship between the number of mutations before and after chemotherapy in paired cancer tissues. (B) scatter plot of the number of mutations before and after chemotherapy; data are presented in log value for pre-therapeutic, post-therapeutic, and common mutations; (C) significant non-synonymous mutations before chemotherapy; (D) significant non-synonymous mutations after chemotherapy; (E) differential mutated genes in paired tissues; (F) lollipop chart for the mutated amino acids of TP53 before and after chemotherapy (the upper one is post-therapeutic, the lower one is pre-therapeutic).

The number of mutations in pre- and post-chemotherapy samples showed a weak linear correlation, although it was not statistically significant (Spearman's correlation = 0.274, *P*=0.129) (Figure 1A). The number of non-synonymous somatic mutations (SNVs + INDELs) was similar before and after chemotherapy, as a median of 215.5 in the primary tumor before chemotherapy and 207.5 after chemotherapy (Wilcoxon signed rank test *P*=0.926) (Figure 1B).

**Figure 2:**
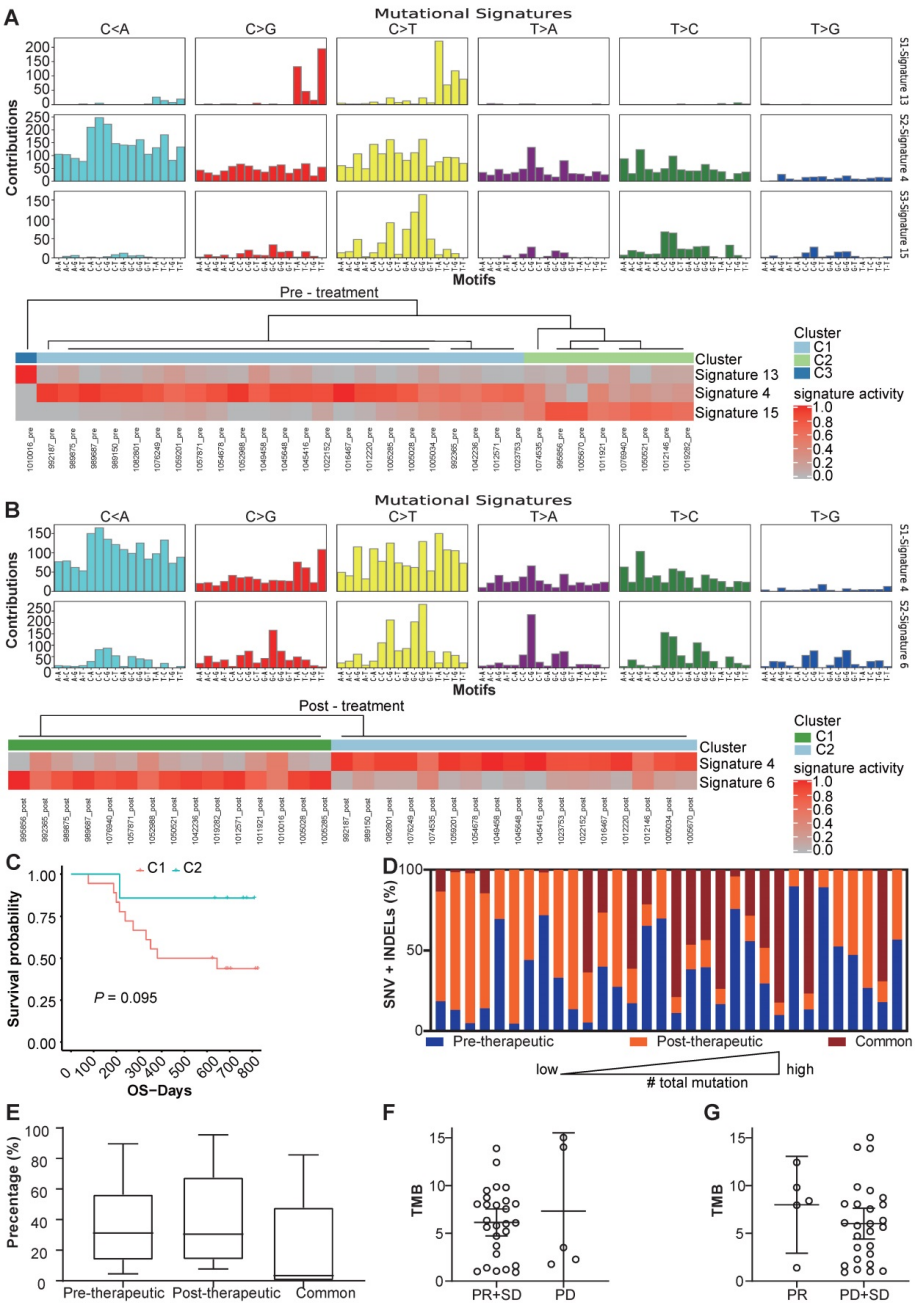
Changes of mutational signature and other mutation characteristics during the chemotherapy. (A) the mutational signature and the clusters of patients before chemotherapy; (B) the mutational signature and clusters of patients after chemotherapy; (C) OS of different clusters before chemotherapy (because only 1 patient was in cluster 3, the cluster 3 was not included in survival analysis); (D) the percentage of unique SNV and INDEL mutations before and after therapy and common mutations of each patients; (E) box and whisker plot for the percentage of unique SNV and INDEL mutations before and after therapy and common mutations for all patients; (F) TMB values for patients with PR/SD and PD (bars represent mean with 95%CI); (G) TMB values for patients with PR and SD/PD (bars represent mean with 95%CI).

We use MutsigCV v1.41 to identify the significant mutations in the samples before and after the chemotherapy respectively (Figure 1C, D). And after comparing significantly mutated genes between per-therapeutic and post-therapeutic, we found the frequency of *TP53* was significantly lower in post-therapeutic samples (Figure 1E). Except for mutation rate, changes of amino acids of the TP53 were also different between pre- and post-therapeutic (Figure 1F). It indicated that tumor cells with different *TP53* mutations might have different sensitivity to chemotherapy. We also found that the mutational signatures of treatment naïve sample were similar to COSMIC signature 4, 13 and 15. While the mutational signatures of post-chemotherapy samples were similar to COSMIC signature 4 and 6 (Figure 2A, B). We divided patients into three groups according to the pre-therapeutic mutational signature and found patients having signature 15 tended to have a better survival although it was not statistically significant which might be due to the small sample size (Figure 2C). By comparing the mutational profile before and after chemotherapy, we found some unique pre-therapeutic and post-therapeutic mutations, and there were also some common mutations that presented before therapy remained after therapy. The ratio of unique mutations before and after therapy and the ratio of common mutations for each patient, were also calculated and the ratio of mutations varied greatly across patients (Figure 2D). The ratio of pre-therapeutic unique mutation and the ratio of post-therapeutic unique mutation was similar (Figure 2E), suggesting that the ratio of post-therapeutic acquired mutations was similar to that of mutations lost due to chemotherapy. The tendency of the ratio of unique mutations after chemotherapy was different in squamous cell carcinoma (LUSC) and adenocarcinoma (LADC), suggesting that chemotherapy might exert distinct effects on LUSC and LADC (Figure S3). We further explored whether the pre-therapeutic tissue TMB is able to predict response to chemotherapy. No significant differences in TMB counts were found between patients who were stable or responded to chemotherapy and those who progressed (Figure 2F), or between the PR and the SD/PD group (Figure 2G), suggesting that TMB was unable to predict response to chemotherapy. Pathway enrichment analysis suggested that the unique mutations present before therapy reflected a variety of functions or pathways (Figure S4A), while the unique mutations after therapy frequently exhibited signal transduction, and negative regulation of canonical Wnt signaling pathways (Figure S4B).

**Figure 3:**
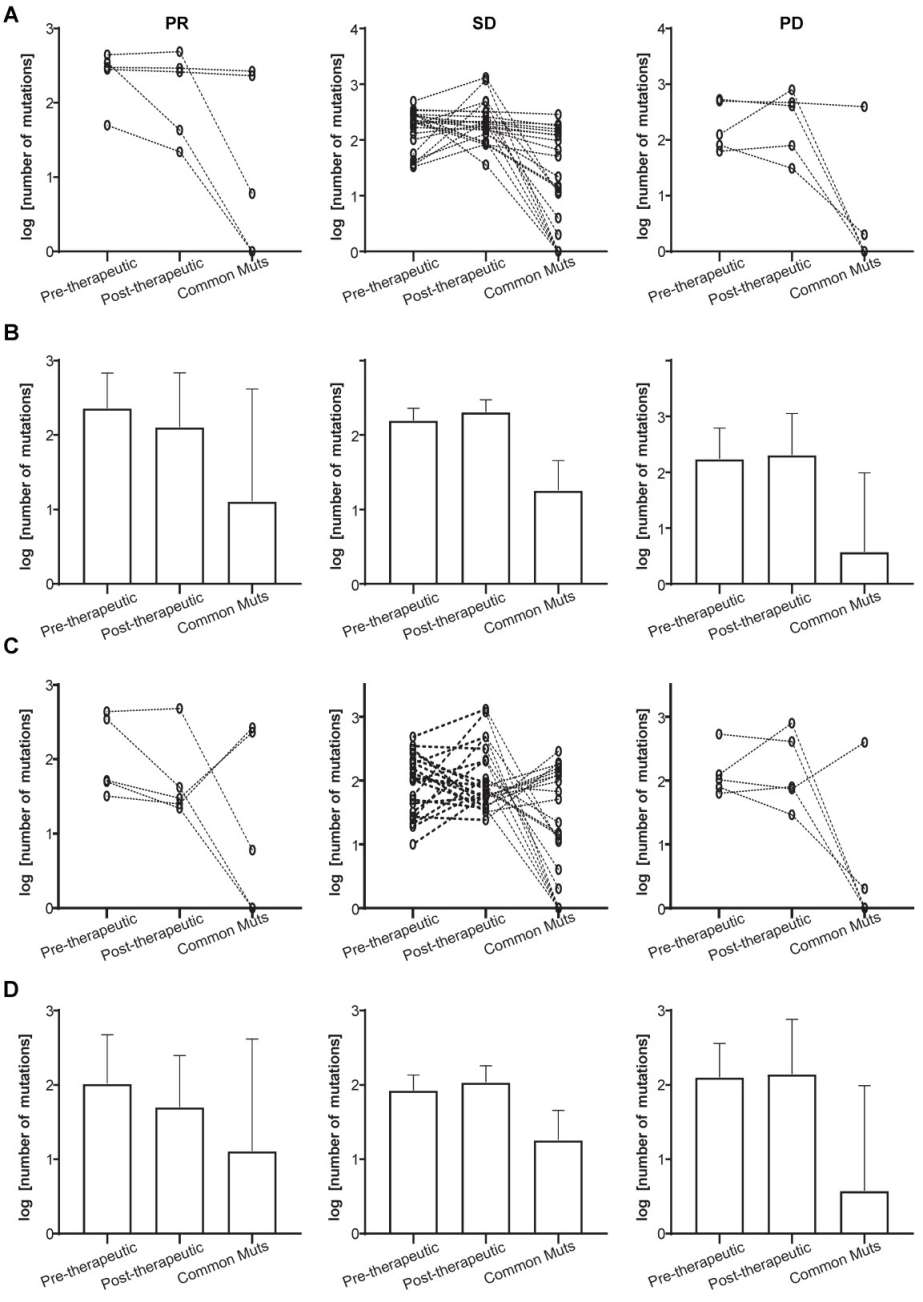
Total and unique mutational changes following first-line chemotherapy in patients with PR, SD, or PD. (A) The slope charts for all mutations before and after chemotherapy, and common mutations in the 3 patient groups; (B) mean values with 95% CI for all mutations before and after chemotherapy, and common mutations in the 3 patient groups; (C) the slope charts for unique mutations before and after chemotherapy, and common mutations in the 3 patient groups; (D) mean values with 95% CI for unique mutations before and after chemotherapy, and common mutations in the 3 patient groups.

We also investigated the mutational changes that must have occurred during chemotherapy in patients attaining PR, and those who showed SD or PD. It can be seen from Figure 3A and 3B that a slightly decrease in the total number of mutations is present in the PR group (the left panels), while the number of common mutations before and after therapy was similar to that in the SD groups (the left and middle panels). The decreasing total number of mutations was not found in the SD and PD group (Figure 3A and 3B, middle and right panels). Since some pre-therapeutic mutations had disappeared after chemotherapy and other mutations appeared, we attempted to unravel the potential relationship between pre- and post-therapeutic unique and common mutations (Figure 3C, 3D). Figure 3C and 3D showed that the number of post-therapeutic unique mutations had decreased in the PR group (left panels). This was not the case in the SD and PD groups. And the number of common mutations of the PR group was similar to that in the SD group (Figure 3C and 3D, left and middle panels).

**Figure 4:**
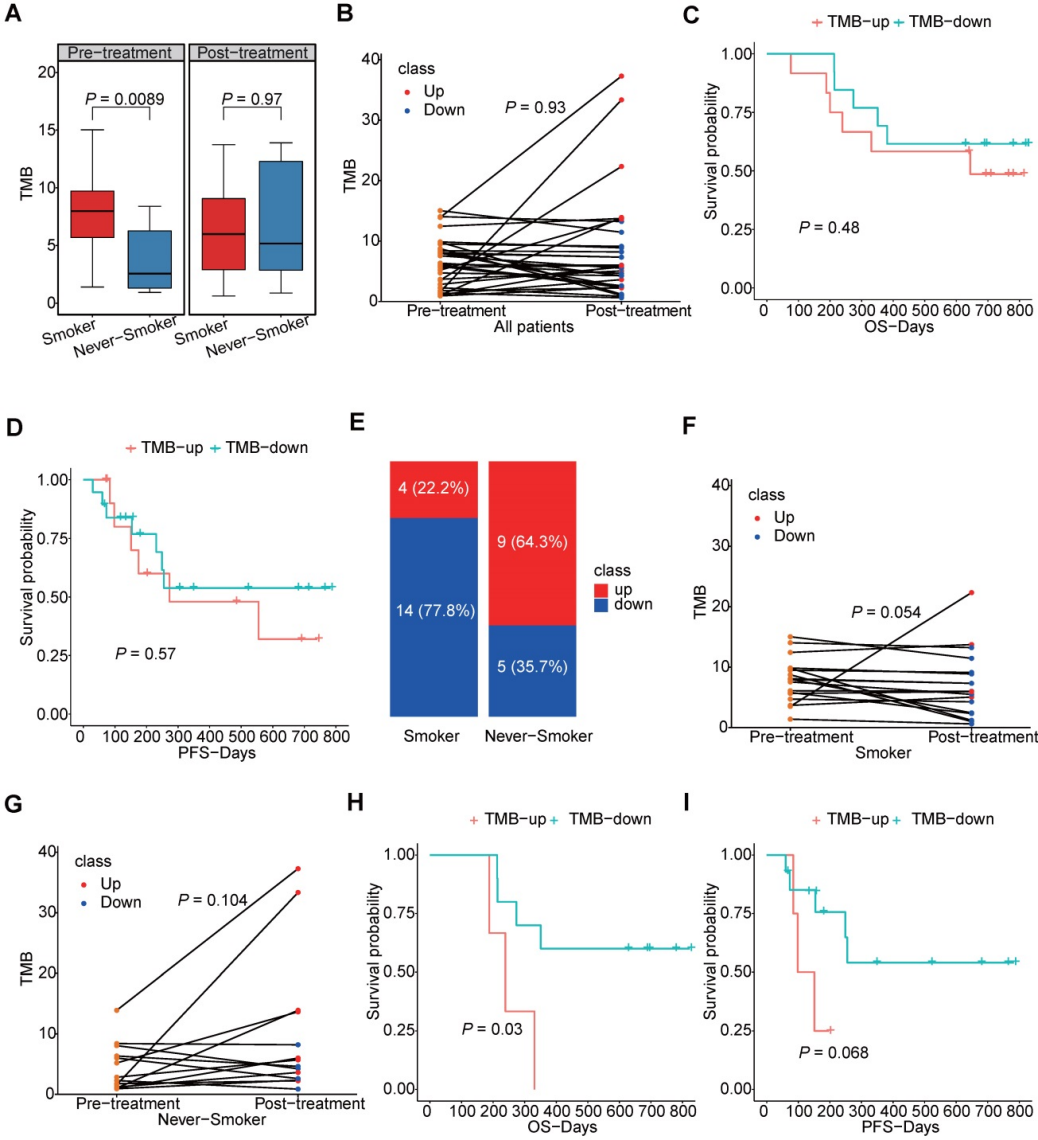
The TMB level in smokers and never-smokers. (A) box plot for TMB values in smokers and never-smokers before and after chemotherapy; (B) the slope chart for the TMB values before and after chemotherapy of each patients; (C, D) the correlation between survival and trend of TMB change; (E) the percentage of TMB-up and TMB-down in smokers and never-smokers respectively; (F, G) the slope chart for TMB values before and after chemotherapy of smokers and never-smokers respectively; (H, I) the correlation between survival and trend of TMB change in smokers.

We further explored the TMB of the samples and its changes induced by chemotherapy. Not surprisingly, we found patients having smoking history would have higher TMB level than those who had never smoked, but we also found this difference no longer existed after chemotherapy (Figure 4A). As shown in Figure 4B, changes of patients TMB were variable among different patients and TMB was not significantly different between pre-chemotherapy and post-chemotherapy samples. Moreover, neither increased TMB nor decreased TMB after chemotherapy showed benefit for overall survival (OS) or progression free survival (PFS) (Figure 4C, D). As smoking had a great impact on the TMB, changes of the TMB were compared separately in smokers and never-smokers. Chemotherapy induced TMB decreasing was at a higher frequency in smokers than that in never-smokers (77.8% vs. 35.7%, *P*=0.03) (Figure 4E). And the TMB level was significantly reduced in smokers after chemotherapy, which was not found in never-smokers (Figure 4F, G). Meanwhile, patients having smoking history who had decreased TMB would have a better survival in both OS and PFS, although it was not statistically significant in PFS (Figure 4H, I). However, this tendency did not occurred in patients who had never smoked (Figure S5). And we also compared mutation frequencies of genes before and after the chemotherapy in smokers and never-smokers. It was found that in smokers, mutational frequencies of 7 genes were significantly decreased, while mutational frequency of *IRX4* was increased (Table 1). It suggested that cells with mutated *ASXL3*, *TP53*, *FSIP2*, *PCDHGA2*, *PTGER1*, *RP1* and *SALL1* might be sensitive to chemotherapy. In never-smokers, we did not find any genes with significantly decreased mutational frequency after chemotherapy, while we found mutational frequencies of 6 genes were increased (Table 2).

**Figure 5:**
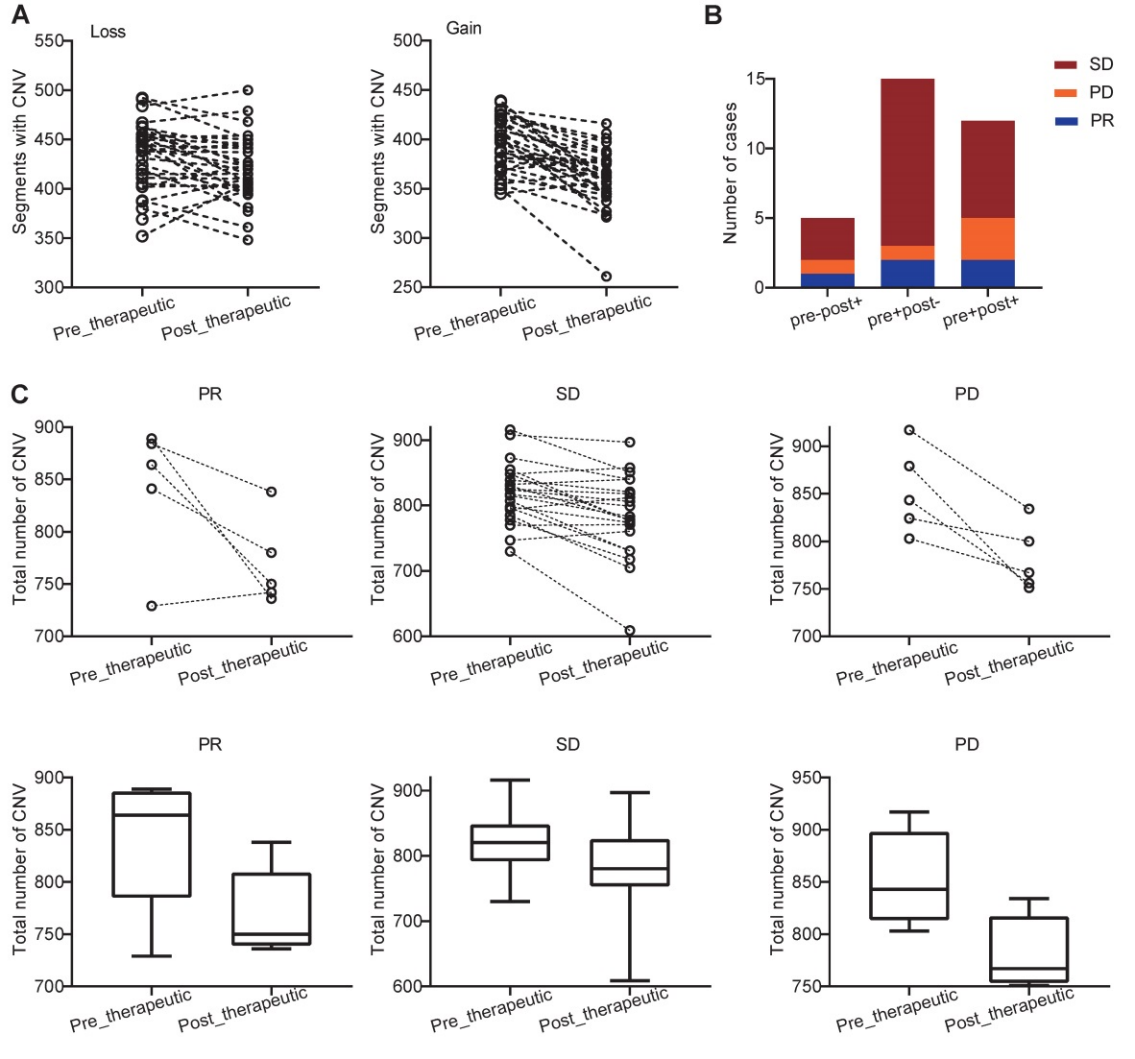
Distribution and change of CNV in patients with PR, SD, and PD. (A) changes of CNVs before and after chemotherapy of each patients; (B) the number of cases with pre-post-, pre+post+, pre+post-, and pre-post+CNV mutations in the PR, SD, and PD groups (because no patients was classified into pre-post- group, the group was not shown in bar plot); (C) the slope chart and, box and whisker plot for the PR, SD, and PD groups before and after chemotherapy.

We further examined how chemotherapy affected the copy number variations (CNVs) in NSCLC patients. We found that both the pre- and post-therapeutic CNVs were distributed across the majority of chromosomes, and the CNVs were varied from patient to patient (Figure S6). At arm-level, we identified 5 significant arm-level amplification and 10 arm-level depletion in pre-therapeutic samples and 5 significant arm-level amplification and 7 arm-level depletion in post-therapeutic samples (Figure S7A). At focal level, the number of significant amplification peaks and deletion peaks was similar in per- and post-therapeutic samples (Figure S7B, C). At segment level, we found that the number of segments with CNV loss and the number of segments with CNV gain were both decreased after chemotherapy (*P_loss_*=0.011, *P_gain_*<0.001, Figure 5A). To further study the details of the CNV changes following chemotherapy, patients were divided into 4 groups: pre-therapeutic CNV negative and post-therapeutic CNV negative (pre-post-), pre-therapeutic CNV positive and post-therapeutic CNV positive (pre+post+), pre-therapeutic CNV positive and post-therapeutic CNV negative (pre+post-), and pre-therapeutic CNV negative and post-therapeutic CNV positive (pre-post+). Figure 5B showed that the number of patients with pre-post-, pre+post+, pre+post-, and pre-post+ CNVs was 0, 12, 15, and 5 respectively and that the majority of patients either had CNV before chemotherapy (pre+post-) or had CNV that was present all the time (pre+post+) (Figure 5B). Further grouping by therapeutic response showed that in only 1 out of 5 PR patients had increased total CNV number after chemotherapy (Figure 5C). A decreasing trend of CNV number was found in the PR group, but because of the small sample size the statistical difference was not reached (Wilcoxon signed rank test, *P*=0.125). In patients with SD, a significant decreased total CNV number was detected (Wilcoxon signed rank test, *P*<0.001). A similar trend was also observed in patients with PD, but statistical significance was at borderline (Wilcoxon signed rank test, *P*=0.063) (Figure 5C).

**Figure 6:**
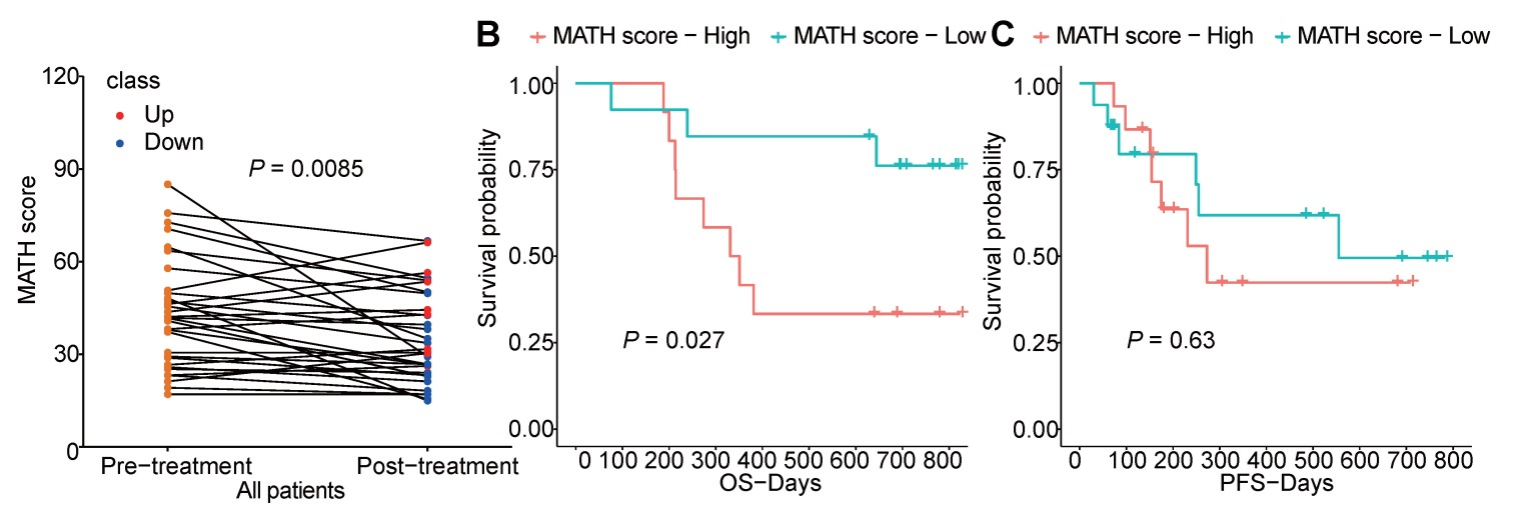
The heterogeneity of tumor before and after chemotherapy. (A) changes of MATH score before and after chemotherapy of each patient; (B, C) survival analysis of pre-therapeutic MATH score.

Furthermore, we analyzed the tumor heterogeneity by calculating MATH scores of samples before and after chemotherapy. Although total non-synonymous somatic mutations did not change after chemotherapy, we found the MATH score was significantly decreased after chemotherapy which indicated that chemotherapy abated the tumor heterogeneity (Figure 6A). Meanwhile, higher pre-therapeutic MATH score had a correlation with shorter OS (*P*=0.027), while this was not detected in PFS (Figure 6B, C).

### Characteristic mutational alterations in NSCLC without TKI-related driver gene mutations

**Figure 7:**
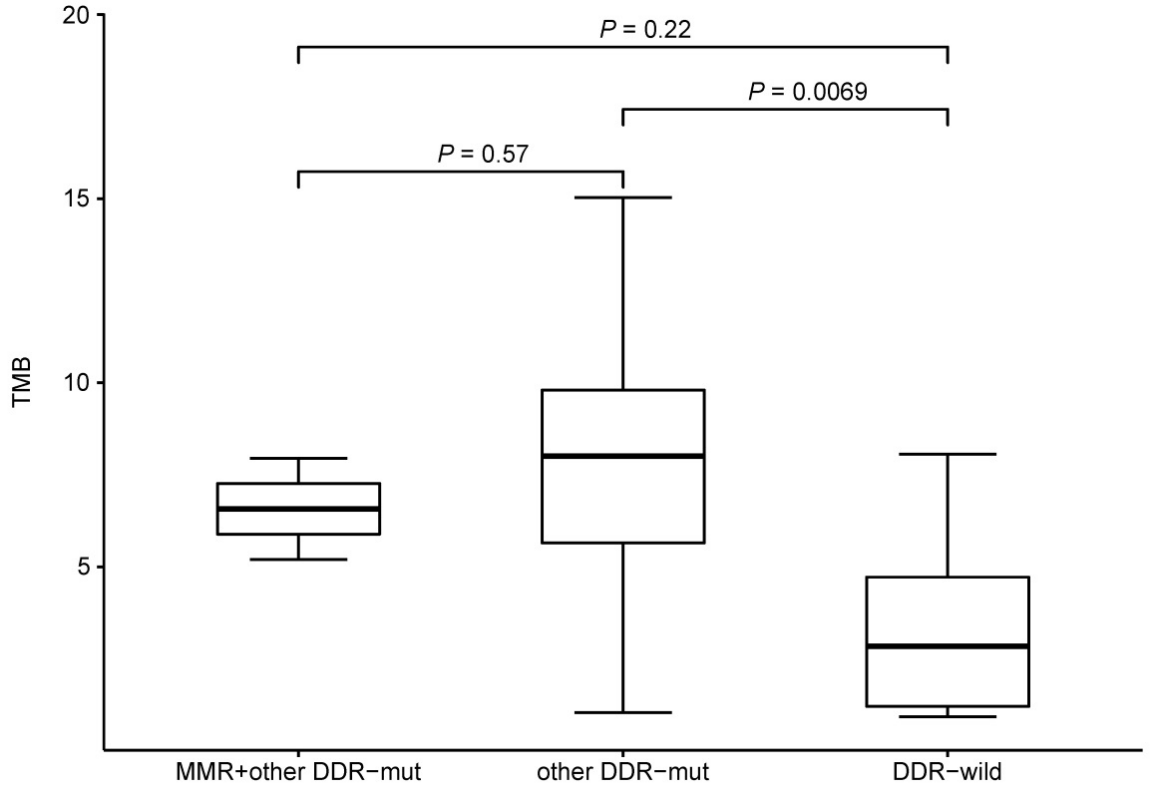
TMB level and mutational status of DNA damage repair related genes.

We also investigated the potential effect of pre-therapeutic DNA damage response (DDR)-relevant mutations on TMB. We compared the TMB between subjects with or without mutations in mismatch repair (MMR)-related genes (*MLH1, MSH2, MSH3, MSH6, MLH3, PMS1, PMS2, MSH4* and* MSH5*) and other DDR-relevant genes (*BRCA1/2, TP53, ATR, CDK12, MRE11A, ATM, PTEN, RAD51, BARD1, BRIP1, CHEK1/2, POLE* and* POLD1*). Comparing to DDR wild-type patients, patients having other DDR-relevant gene mutations would have higher TMB level (Figure 7). Only 2 patient had a *MLH3* mutation (pre-therapeutic TMB=5.2, 7.95) while no mutations were identified in other MMR-related genes. No mutations were found in *ATM, RAD51, BARD1, BRIP1, CHEK1/2,* and* POLD1*. While patients with *PTEN* or *PLOE* mutation would have a higher TMB level (*P_PTEN_*= 0.03, *P_PLOE_*= 0.02). No significant difference (*P*>0.05) was found between subjects with or without mutations in *BRCA1/2, TP53, ATR, CDK12,* or* PMS1*. These results suggested that the some mutated genes of DDR pathway might affect TMB.

We also compared the ratio of mutations from 1574 genes in LADC and LUSC (Table S2), and statistically significant differences (*P*<0.05) were found in 4 genes: *PCDH11X, MYO16, HERC2* and* USH2A*. When *P*<0.10 was regarded as the threshold of statistical significance, another 16 genes showed statistical differences: *NFE2L2, POUSF3, NYAP2, ATR, FAT1, CSMD1, SPATA6L, LRP2, NDST4, ADGRV1, ANKRD30A, OTOF, ROS1, ADGRL1, DLGAP2*, and *DYNC2H1*. Due to the limited number of patients in this study, our data should be interpreted with caution. In that context, it is important to note that certain genes showed a different mutational status in LADC and LUSC. It is not excluded that these differences that might become a clue to new therapeutic approaches in the future.

The correlation between mutational status, clinicopathological factors, and chemotherapy responses was also assessed. It can be seen from data given in Table S3. And from all genes with computable mutation frequency, 32 genes showed a statistically significant difference between disease-controlled and disease-progressed, suggesting that mutations of these 32 genes might predict therapeutic response.

### Signaling pathways and neoantigens (NeoAgs) related to immunotherapeutic responses were altered by first-line chemotherapy

**Figure 8:**
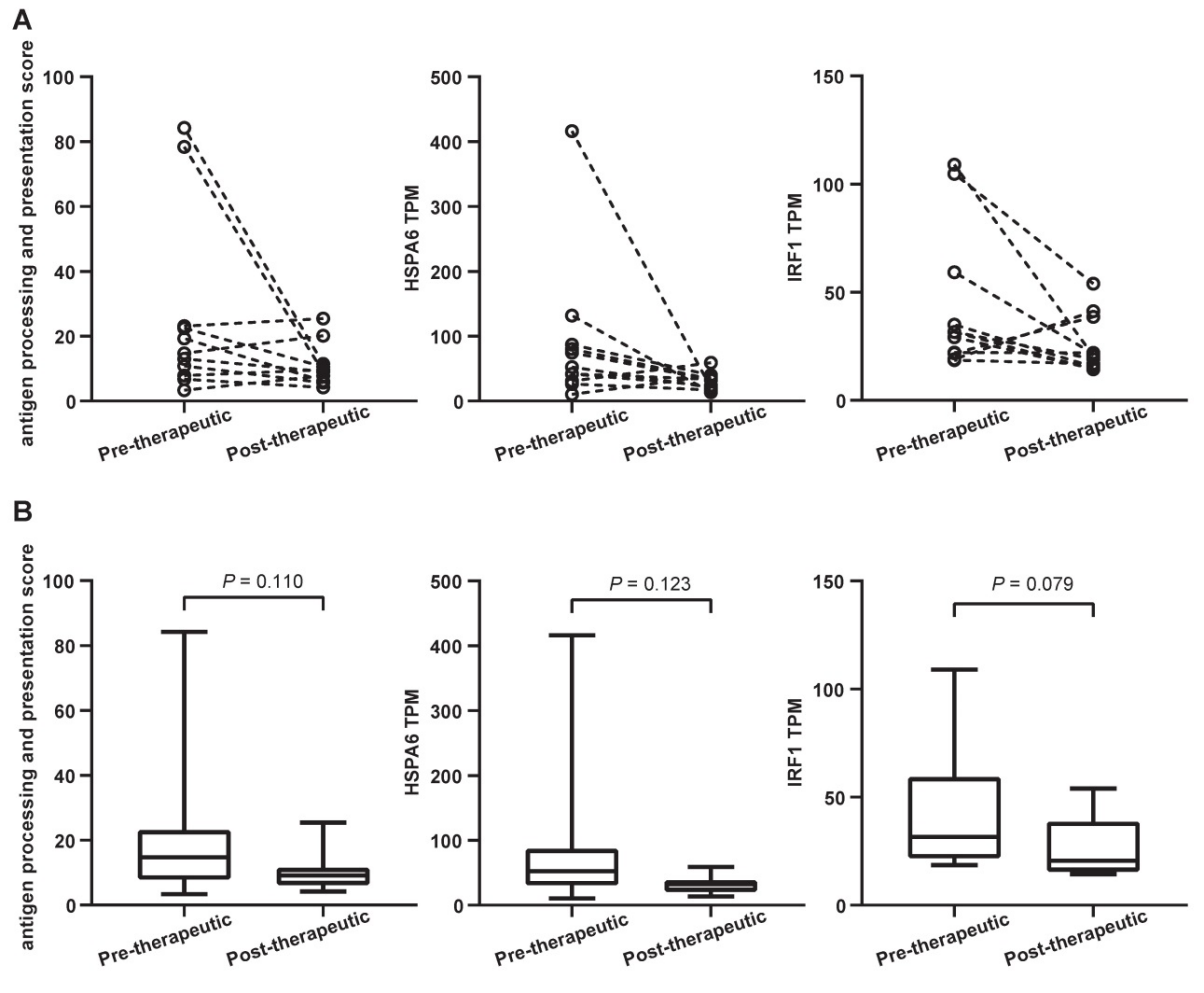
Antigen processing and presentation changes following the first-line chemotherapy. (A) the slope chart for antigen processing and presentation score (APPS), *HSPA6* gene transcripts per million reads (TPM) and *IRF1* gene TPM; (B) the box and whisper plot for APPS, *HSPA6* gene TPM and *IRF1* gene TPM.

A considerable number of NSCLC patients will develop resistance after chemotherapy and be considered candidates for (second-line) immunotherapy. It is known that antigen processing and presentation are critical for anti-PD-1/ programmed death ligand 1 (PD-L1) therapies. To determine whether chemotherapy is able to affect these biological processes, the antigen processing and presentation scores were calculated for both pre-therapeutic and post-therapeutic tumor samples. They were based on the average mRNA levels of *HLA-DMB, HLA-DQA1, HLA-DQA2, HSPA6, KIR2DL3, KIR3DL1,* and *KLRC1*. Results from 11 patients showed that the score decreased after chemotherapy although it did not reach the statistical significance (*P*=0.110) (Figure 8A and 8B, left panels). The mRNA level of all genes except* KIR3DL1* exhibited a decreasing trend, including 1 representative gene, *HSPA6* (Figure 8A and 8B, middle panels). The expression of *HSPA6* (presented as transcripts per million reads or TPM) decreased from a mean value of 90.3 before chemotherapy to 30.3 after chemotherapy. Interestingly, a typical IFN-γ-related gene called *IRF1* tended to be down-regulated following chemotherapy, but the significance was at borderline (Figure 8A and 8B, right panels).

**Figure 9:**
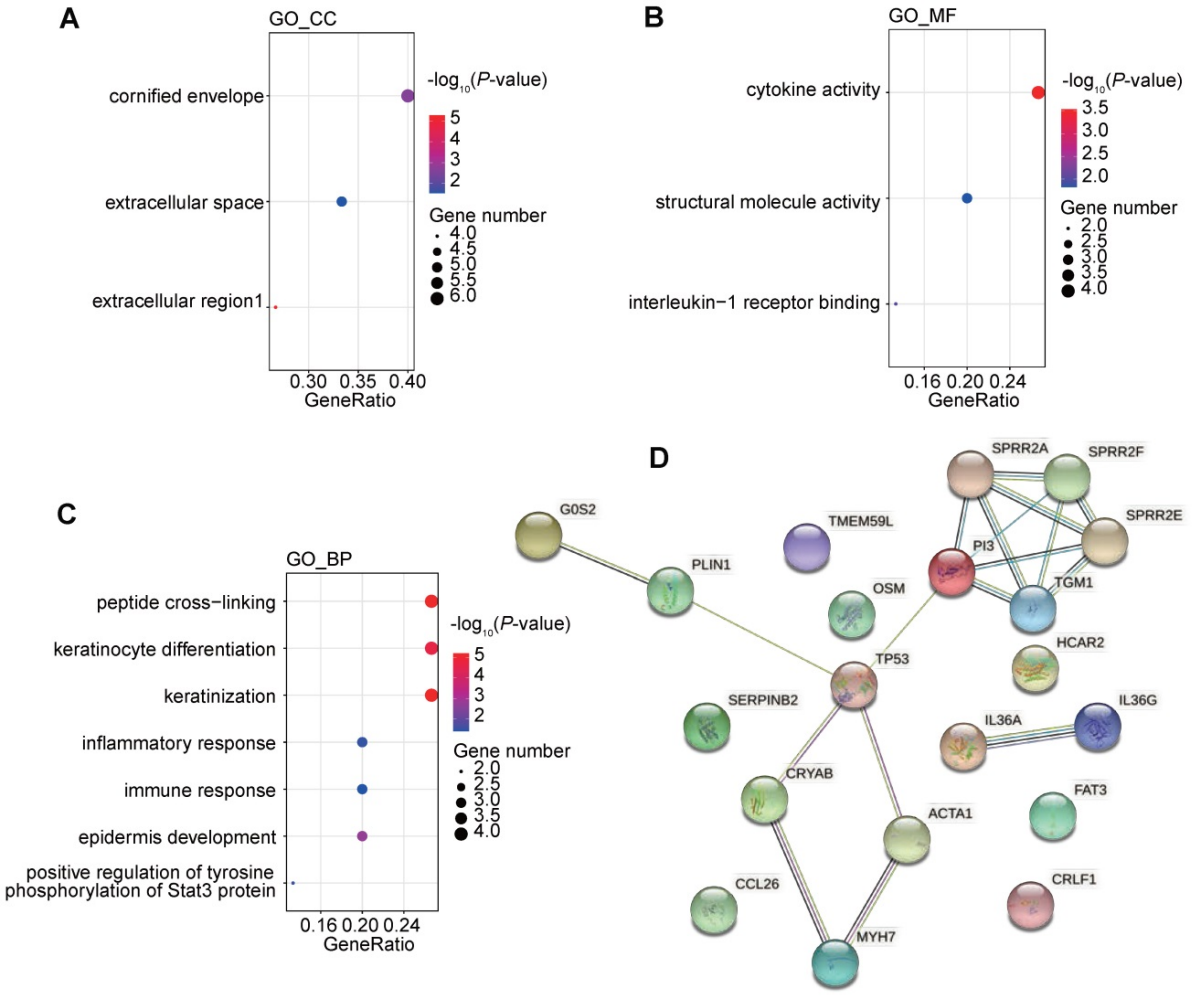
Functional enrichment of differentially expressed genes after chemotherapy and interactions between changes of genome and transcriptome induced by chemotherapy. (A-C) GO analysis of down regulated genes following the first-line chemotherapy; (D) protein-protein interactions among differentially expressed genes and differentially mutated genes following the first-line chemotherapy.

We further investigated the corresponding pathways of the down-regulated genes by GO and KEGG pathway enrichment analysis (Figure 9A-C). It was found that the down-regulated genes were strongly enriched in immune-related biological processes or molecule function, including inflammatory response (GO_BP), immune response (GO_BP), positive regulation of tyrosine phosphorylation of Stat3 protein (GO_BP), cytokine activity (GO_MF), and interleukine-1 receptor binding (GO_MF). These alterations indicated that the chemotherapy might negatively regulate the immune status and might impair the efficacy of ICIs.

We further tried to analyze the potential correlation between genomic changes and transcriptomic changes after chemotherapy. Transcription factor analysis showed that the down-regulated gene, *CRYAB*, could be regulated by differentially mutated gene, *TP53*. Protein-protein interaction (PPI) also suggested a complex interactions among proteins coded by differentially mutated and expression genes. And interactions between *TP53* and 4 differentially expressed genes were detected (Figure 9D). It indicated that the genomic changes caused by chemotherapy might have a profound impact on gene expression profile after chemotherapy.

**Figure 10:**
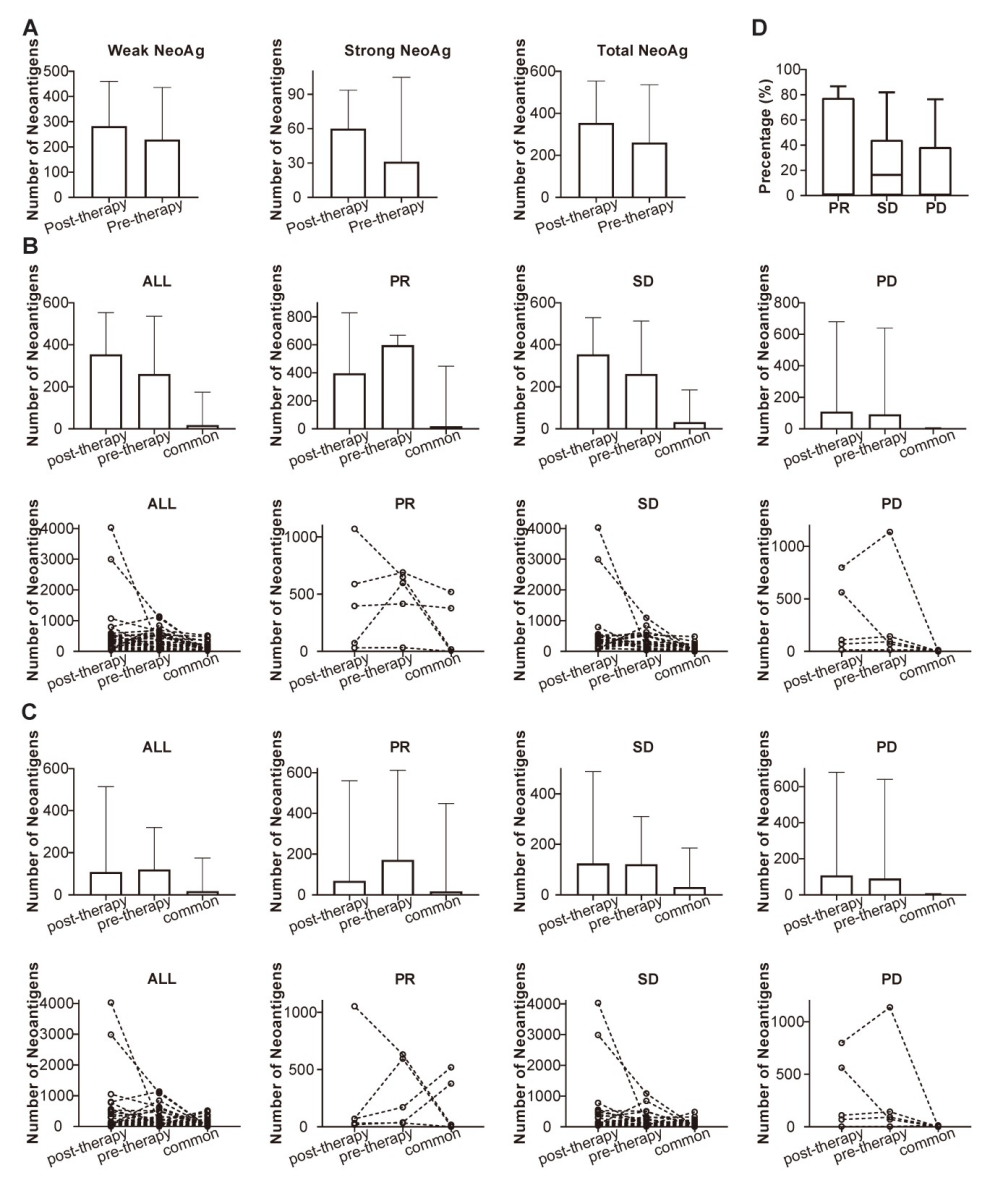
Comparison of the number of pre-, post-therapeutic and common NeoAgs. (A) The number of total, strong and weak NeoAgs before and after therapy; (B) the number of total pre- and post-therapeutic and common NeoAgs in patients with PR, SD and PD; (C) the number of unique pre- and post-therapeutic and common NeoAgs in patients with PR, SD and PD; (D) comparison of the percentage of common NeoAgs in patients with PR, SD and PD.

NeoAgs were predicted through the mutational data obtained from WES, and the pre-therapeutic and post-therapeutic status of NeoAgs were determined and compared. It can be observed from Figure 10A that the number of NeoAgs did not change significantly after chemotherapy no matter total NeoAgs, or strong NeoAgs or weak NeoAgs was examined. The changes in NeoAgs were investigated in detail, as shown in Figure 10B. The total number of pre- and post-therapeutic NeoAgs and the common NeoAgs in patients with PR, SD or PD were compared in slope chart and bar plot. It appeared that the number of post-therapeutic NeoAgs show a trend of increase in SD and PD group, while it exhibited a trend of decrease in the PR group although it was not statistically different (*P*=0.438). We further studied the change of pre- and post-therapeutic unique NeoAgs. Figure 10C shows that the number of unique post-therapeutic NeoAgs also exhibited a trend of decrease in the PR group (*P*=0.438), while no such change was observed with the SD and PD group. We finally compared the percentage of common NeoAgs in PR, SD, and PD groups. Figure 10D shows that patients with SD exhibited much higher percentage of common NeoAgs than the PD group, suggesting that the status of NeoAgs changed substantially when cancer lesions were progressed, while remained relatively stable when the lesions were stable.

## Discussion

### The influence of the first-line chemotherapy on mutation spectrum in NSCLC patients without TKI-related driver gene mutations

In order to optimally detect mutations before and after chemotherapy, we collected both pre- and post-therapeutic tissues and used WES to determine the mutation spectrum and TMB (WES is the gold standard test for TMB). We used transcriptome sequencing to obtain complete gene expression information related to chemotherapy response. In the whole cohort, the total non-synonymous mutation was similar before and after chemotherapy. In PR group, all mutations, including unique mutations, decreased after chemotherapy. This suggests that the ratio of unique mutations to all mutations may be constant. Furthermore, it was interesting that common mutation was low in the PD group, while it was relatively high in the PR and SD group. This suggested that when lesions progressed, the mutation spectrum would have changed significantly, with common mutations reduced. The mutation spectrum turned out to be more unstable in patients with progressed disease including common mutations. We also found that the mutational signatures altered after chemotherapy, which suggested that chemotherapy did change the genomic characteristics of tumors. Moreover, pre-therapeutic mutational signature showed an association with survival of the patients. In the whole cohort, we did not find significant changes of TMB after chemotherapy and the change of TMB was not associated with neither OS nor PFS. However, we found a significantly decreased TMB after chemotherapy in smokers. Furthermore, only in patients who had smoking history, decreased TMB would indicated a better survival of chemotherapy. We also found that high intratumoral heterogeneity was associated with poor survival, which was consistent with pervious studies 26, 27. Moreover, analysis showed a decreased the MATH score after chemotherapy which indicated that the chemotherapy could reduce the clone diversity of the tumor. Further analysis of post-therapeutic tumor tissues might give us more information about tumor evolution under chemotherapy stress.

Pathway enrichment analyses showed that pre-therapeutic mutations did not exhibit specific signaling pathways beyond those previously reported in NSCLC, while pathways affected by chemotherapy mainly included signal transduction, intracellular signal transduction, and negative regulation of canonical Wnt signaling pathway. These suggested that cancer cells might aberrantly activate some signaling pathways through genomic changes induced by chemotherapy to survive under the pressure of chemotherapy. Mutations in suppressive factors of Wnt signaling pathway might cause the activation of the Wnt pathway, an oncogenic pathway, of cancer cells 28. Moreover, studies had demonstrated that Wnt pathway had strong correlation with the modulation of tumor immune microenvironment 29. It suggested that aberrant activated Wnt pathway would promote an exclude immune phenotype which was unfavorable for the efficacy of ICIs 30, 31. Meanwhile, Wnt inhibitor had shown its role in enhancing the efficacy of ICIs 32. These observations suggested that both tumor intrinsic characteristics and tumor microenvironment changed profoundly after chemotherapy. And the change of microenvironment might be unfavorable for latter ICIs.

CNV analysis found that, in all response groups, a trend of decreased CNV was detected. However, only in SD group the decreasing was statistical significant. It might be due to the small sample size of our study. Further analyses of genes involved in CNVs showed that the deletion of *CD274*,* CDKN2A* and *PTEN* in some patients after chemotherapy. *CD274* encoded PD-L1 which was one of the target for ICI and studies had suggested that patients with *CD274* amplification would have better immunotherapy outcomes 33, 34. Current research also found that deletion of *CDKN2A* and alteration of *PTEN* increase the resistance to ICIs 35, 36. *NOTCH2* was amplified in some patients before and after chemotherapy. Study showed that deleterious mutation of *NOTCH* indicated a better outcome of ICIs 37. Further studies on these CNV markers are needed to clarify their roles in predicting therapeutic response and prognosis.

### Potential impact of first-line chemotherapy on the therapeutic response to subsequent immunotherapy

Therapy with PD-1 inhibitors such as pembrolizumab and nivolumab have achieved great success in treating advanced NSCLC as both first- and second-line therapy 19, 38-41. However, only a minority of patients responds to therapy. One of the strategies to address the problem of patient selection for immunotherapy is research into biomarkers 12 that are able to accurately identify sensitive patients. KEYNOTE-024 demonstrated that pembrolizumab was associated with significantly longer PFS and OS in advanced NSCLC patients with a PD-L1 (tumor) score ≥ 50% 19, 42, while the Checkmate-026 study reported that nivolumab failed to show any benefit compared with standard chemotherapy based on PD-L1 expression ≥ 5%. Previous studies have suggested that TMB has the capacity to become a better predictor of response to immunotherapy than PD-L1 expression 43. Other predictive biomarkers, such as IFN-γ-related gene expression, antigen presentation, and chemokine expression, have also shown to correlate with clinical responses to immunotherapy 44.

Although a substantial number of studies have focused on biomarkers potentially predicting response to immunotherapy, few have also explored the impact of chemotherapy on subsequent immunotherapy. A meta-analysis including 20,013 patients suggested that no difference was found in the OS between first-line and multiple-line targeted therapy, but a difference was found between first and multiple-line immunotherapy 18. Other studies have confirmed that the response rate to immunotherapy is less favorable if used after chemotherapy 20, 45. PFS following first-line pembrolizumab was 12.9 months, while the PFS of the immunotherapy, which was applied after chemotherapy, was only 4.2 months 19.

Antigen processing and presentation play an important role in immunotherapy and are regarded as key factors for the efficient killing of tumor cells by immune cells. We observed the decrease in antigen processing/presentation following chemotherapy. Although the total number of neoantigens showed a slightly increasing trend after chemotherapy, down-regulated antigen processing and presentation made them could not be recognized by immune system and facilitated the immune escape. It has been shown that the INF-γ signaling pathway regulates the expression of PD-L1 46, 47, and is closely correlated with the efficacy of immunotherapy 19. Some studies have reported that the high expression of IFN-γ-related genes is correlated with the improved efficacy of immunotherapy 44. Patients with a high expression of IRF1 exhibited favorable PFS in metastatic melanoma 48. Moreover, it has been reported that patients with a high expression of MHC-II complex exhibited better OS and PFS when receiving anti-PD-1/PD-L1 treatment 49. Since the expression of IRF1 and MHC protein complex and IFN-γ-related genes is involved in the presentation of tumor antigens 49, down-regulation of their expression might be one of the mechanisms for tumor immune escape. More interestingly, our study suggests that chemotherapy is able to inhibit the expression of these genes (leading to significant down-regulation of lymphocyte activation, IFN-γ, and chemokine-related signaling pathways) and the presentation of NeoAgs, and also indicates that chemotherapy may have multiple inhibitory effects on subsequent immunotherapy which in practice may translate into resistance.

## Conclusions

We systematically explored the genomic and transcriptomic consequences of chemotherapy in paired biopsy samples of 32 driver mutation-negative NSCLC patients and assessed the potential influence of genomic and transcriptomic changes on immunotherapy response. Reduction of mutation burden and transcriptional levels of key antigen processing and presentation-related genes were noticed after chemotherapy, likely reflecting mutational and transcriptional inhibitory effects of chemotherapy. These alterations might promote a tumor microenvironment resistant to subsequent immunotherapy. Our observations support the use of immunotherapy in advanced/metastatic NSCLC in a first-line as an opposed to a second-line setting.

## Supplementary Material

Supplementary figures and tables.

## Figures and Tables

**Table 1:** Differentially mutated genes before and after chemotherapy in smokers.

Symbol	disappeared	acquired	retained	*P* value	comparing to per-therapy
ASXL3	5	0	3	0.025	decreased
TP53	5	0	9	0.025	decreased
FSIP2	4	0	0	0.046	decreased
IRX4	0	4	0	0.046	increased
PCDHGA2	4	0	0	0.046	decreased
PTGER1	4	0	0	0.046	decreased
RP1	4	0	2	0.046	decreased
SALL1	4	0	0	0.046	decreased

**Table 2:** Differentially mutated genes before and after chemotherapy in never-smokers.

Symbol	disappeared	acquired	retained	*P* value	comparing to per-therapy
RYR1	0	7	0	0.008	increased
ABCA2	0	4	0	0.046	increased
COL22A1	0	4	0	0.046	increased
TTN	0	4	3	0.046	increased
VPS13B	0	4	1	0.046	increased
ZSWIM6	0	4	0	0.046	increased

## Abbreviations

mRNA: messager ribonucleic acid
DNA: deoxyribonucleic acid
ALK: anaplastic lymphoma kinase
CNVs: copy number variations
DDR: DNA damage response
ECM: extracellular matrix
EGFR: epidermal growth factor receptor
GO: Gene Ontology
ICIs: Immune checkpoint inhibitors
KEGG: Kyoto Encyclopedia of Genes and Genomes
LADC: lung adenocarcinoma
LUSC: lung squamous cell carcinoma
MMR: mismatch repair
NeoAgs: neoantigens
NGS: next-generation sequencing
NSCLC: non-small cell lung cancer
OS: overall survival
PBL: peripheral blood lymphocyte
PCR: polymerase chain reaction
PD: progression of disease
PD-1: programmed death-1
PD-L1: programmed death ligand 1
PFS: progression-free survival
PR: partial response
SD: stable disease
TIME: tumor immune microenvironment
TKI: tyrosine kinase inhibitor
TMB: tumor mutational burden
TPM: transcripts per million reads
WES: whole-exome sequencing
