# Supplementary material for: Genomic and transcriptional alterations in first-line chemotherapy exert a potentially unfavorable influence on subsequent immunotherapy in NSCLC
Source: Theranostics. 2021 May 13;11(14):7092–109. doi: 10.7150/thno.58039 (PMC8171101; doi:10.7150/thno.58039)
Supplement: Supplementary file 1 — Supplementary figures and tables. [file thnov11p7092s1.pdf]

Table S1 Clinical and pathological characteristics of patients

Sample_ID	Age	Stage	Smoking	Sex	Subtype	response
989150	80	Stage IV	Never-Smoker	Male	LADC	SD
989875	63	Stage III	Smoker	Male	LUSC	PR
989687	70	Stage IV	Smoker	Male	LADC	SD
992365	76	Stage IV	Never-Smoker	Male	LUSC	SD
992187	60	Stage II	Never-Smoker	Male	LUSC	PR
995856	62	Stage III	Never-Smoker	Female	LADC	SD
1005670	51	Stage III	Smoker	Male	LADC	SD
1005028	58	Stage IV	Smoker	Male	LUSC	PR
1005034	57	Stage III	Smoker	Male	LUSC	SD
1005285	71	Stage III	Smoker	Male	LADC	PD
1010016	73	Stage IV	Smoker	Male	LADC	SD
1011921	58	Stage IV	Smoker	Male	LUSC	PR
1012220	65	Stage III	Smoker	Male	LADC	SD
1057871	56	Stage IV	Never-Smoker	Male	LUSC	PD
1012571	22	Stage IV	Never-Smoker	Male	LUSC	PD
1019282	50	Stage III	Never-Smoker	Female	LADC	SD
1023753	72	Stage III	Never-Smoker	Male	NSCLC	SD
1042236	68	Stage IV	Never-Smoker	Male	LUSC	SD
1016467	57	Stage IV	Smoker	Male	NSCLC	PD
1012146	50	Stage IV	Never-Smoker	Female	LADC	SD
1045648	73	Stage IV	Smoker	Male	LUSC	PR
1049458	75	Stage IV	Smoker	Male	LADC	PD
1045416	66	Stage III	Smoker	Male	LUSC	SD
1022152	65	Stage III	Never-Smoker	Male	LADC	SD
1050521	40	Stage III	Never-Smoker	Female	LADC	SD
1054678	67	Stage III	Smoker	Male	LADC	SD
1059201	73	Stage IV	Smoker	Male	LADC	SD
1074535	67	Stage IV	Smoker	Male	LADC	SD
1052988	74	Stage IV	Smoker	Male	LUSC	SD
1076940	62	Stage IV	Never-Smoker	Female	LADC	SD
1076249	70	Stage IV	Never-Smoker	Female	LADC	SD
1082801	73	Stage I	Smoker	Male	LUSC	SD

Table S2 Differentially mutant genes between LUSC and LADC

Gene	LADC_ mut	LADC_ WT	LUSC_ mut	LUSC_ WT	Freq_ LADC (%)	Freq_ LUSC (%)	*P*_value
PCDH11X	0	17	5	8	0.00	38.46	0.009
MYO16	0	17	4	9	0.00	30.77	0.026
HERC2	0	17	4	9	0.00	30.77	0.026
USH2A	2	15	6	7	11.76	46.15	0.049
NFE2L2	1	16	5	8	5.88	38.46	0.061
POU3F3	0	17	3	10	0.00	23.08	0.070
NYAP2	0	17	3	10	0.00	23.08	0.070
ATR	0	17	3	10	0.00	23.08	0.070
FAT1	0	17	3	10	0.00	23.08	0.070
CSMD1	0	17	3	10	0.00	23.08	0.070
SPATA6L	0	17	3	10	0.00	23.08	0.070
LRP2	0	17	3	10	0.00	23.08	0.070
NDST4	0	17	3	10	0.00	23.08	0.070
ADGRV1	0	17	3	10	0.00	23.08	0.070
ANKRD30A	0	17	3	10	0.00	23.08	0.070
OTOF	0	17	3	10	0.00	23.08	0.070
ROS1	0	17	3	10	0.00	23.08	0.070
ADGRL1	0	17	3	10	0.00	23.08	0.070
DLGAP2	0	17	3	10	0.00	23.08	0.070
DYNC2H1	0	17	3	10	0.00	23.08	0.070

Table S3 Differentially mutant genes between PR/SD and PD patients

Gene	PR/SD_ mut	PR/SD_ WT	PD_ mut	PD_ WT	freq_PR/SD (%)	freq_PD (%)	*P*_value
FZD10	0	27	3	2	0.00	60.00	0.002
RBM25	0	27	3	2	0.00	60.00	0.002
RIMS2	1	26	3	2	3.70	60.00	0.008
OTOG	2	25	3	2	7.41	60.00	0.018
ZNF518B	0	27	2	3	0.00	40.00	0.020
UGGT2	0	27	2	3	0.00	40.00	0.020
CBX8	0	27	2	3	0.00	40.00	0.020
KEAP1	0	27	2	3	0.00	40.00	0.020
SOX12	0	27	2	3	0.00	40.00	0.020
FOXB2	0	27	2	3	0.00	40.00	0.020
NEB	0	27	2	3	0.00	40.00	0.020
SLC6A1	0	27	2	3	0.00	40.00	0.020
CCDC96	0	27	2	3	0.00	40.00	0.020
TIFA	0	27	2	3	0.00	40.00	0.020
ADCY2	0	27	2	3	0.00	40.00	0.020
PDZRN4	0	27	2	3	0.00	40.00	0.020
CAPS2	0	27	2	3	0.00	40.00	0.020
SHISA2	0	27	2	3	0.00	40.00	0.020
NRDE2	0	27	2	3	0.00	40.00	0.020
RTL1	0	27	2	3	0.00	40.00	0.020
GTF3C1	0	27	2	3	0.00	40.00	0.020
BCAR1	0	27	2	3	0.00	40.00	0.020
NFATC1	0	27	2	3	0.00	40.00	0.020
LANCL3	0	27	2	3	0.00	40.00	0.020
ATP7A	0	27	2	3	0.00	40.00	0.020
NPR1	0	27	2	3	0.00	40.00	0.020
TDP2	0	27	2	3	0.00	40.00	0.020
CNGA4	0	27	2	3	0.00	40.00	0.020
EPS8	0	27	2	3	0.00	40.00	0.020
SSH1	0	27	2	3	0.00	40.00	0.020
TCF4	0	27	2	3	0.00	40.00	0.020
KRAS	3	24	3	2	11.11	60.00	0.034

Figure S1. Illustration of demographic information of all patients involved in this study. Information on sex, age, smoking history, cancer subtype, cancer stage, and therapeutic response of all patients are shown by bars with distinct colors. Sampling conditions in WES (DNA) is also indicated. LADC = lung adenocarcinoma; LUSC = lung squamous cell carcinoma.

Figure S2. Flowchart showing the procedure of sampling, targeted NGS, and data analysis in this study. Lung cancer tissue samples were collected before and after chemotherapy and were examined by WES and transcriptome sequencing. Mutational change, TMB, antigen presentation, and their correlation with therapeutic response were analyzed.

Figure S3. The status of unique mutations and common mutations before and after chemotherapy. The ratio of unique and common mutations is shown for each patient for LADC and LUSC in the left panel, and the statistics for these is shown in the right panels. A significantly lower percentage was found in post-therapeutic unique mutations in LUSC while this was not observed with LADC.

Figure S4. Pathway enrichment analysis on mutations before and after chemotherapy. (A) GO and KEGG enrichment analysis on mutations before chemotherapy; (B) GO and KEGG enrichment analysis on mutations after chemotherapy

Figure S5. The correlation between the trend of TMB change and patients survival of never-smoking patients.

Figure S6. The distribution of CNVs across each chromosome of each patients. (A) the CNVs before chemotherapy; (B) the CNVs after chemotherapy.

Figure S7. The distribution of CNVs at arm and focal level. (A) the distribution of CNVs at the arm level (red represents the amplification, blue represents the deletion; chromosome having significant amplification was marked in red, having significant deletion was marked in blue and having both significant amplification and deletion was marked in green); (B) the distribution of pre-therapeutic CNVs at the focal level (red represents the amplification, blue represents the deletion); (C) the distribution of post-therapeutic CNVs at the focal level (red represents the amplification, blue represents the deletion).

Figure S1

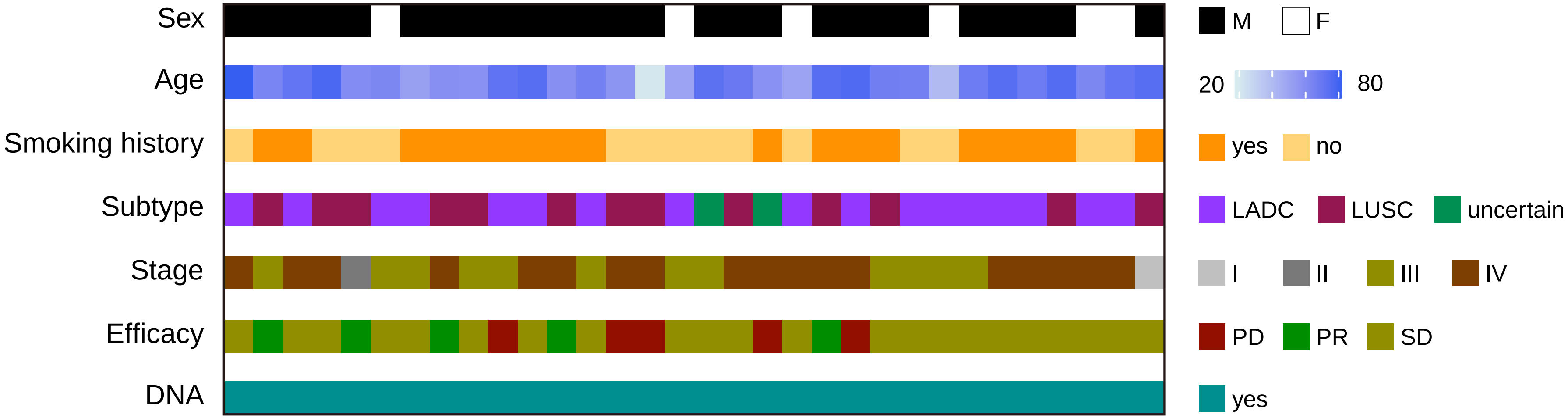
Sex
Age
Smoking history
Subtype
Stage
Efficacy
DNA
M
F
20
80
yes
no
LADC
LUSC
uncertain
I
II
III
IV
PD
PR
SD
yes

Figure S2

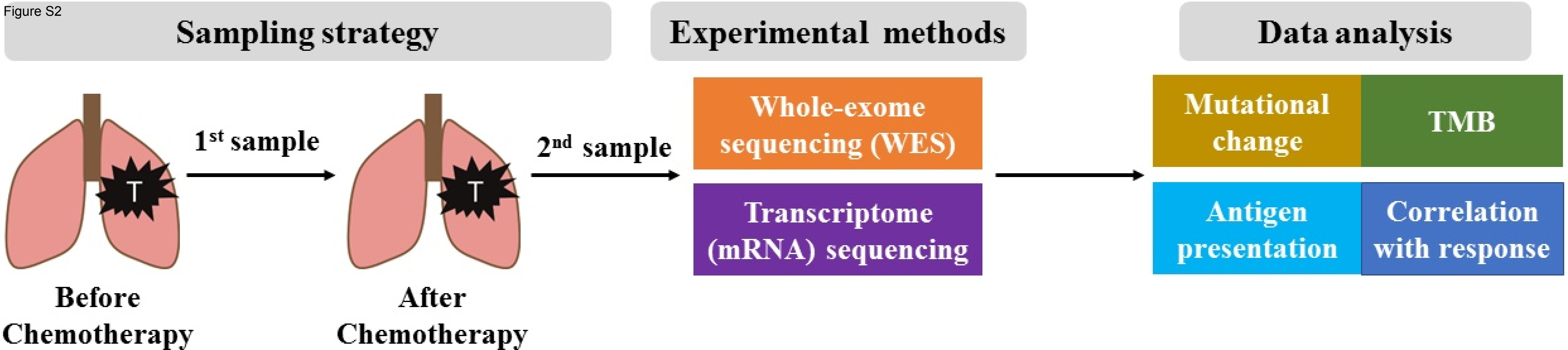
Sampling strategy
Experimental methods
Data analysis
1st sample
2nd sample
T
T
Before Chemotherapy
After Chemotherapy
Whole-exome sequencing (WES)
Transcriptome (mRNA) sequencing
Mutational change
TMB
Antigen presentation
Correlation with response

Figure S3

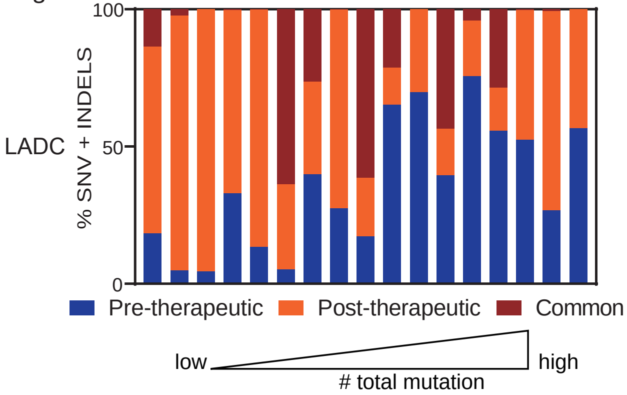
LADC
% SNV + INDELS
100
50
0
Pre-therapeutic
Post-therapeutic
Common
low
high
total mutation

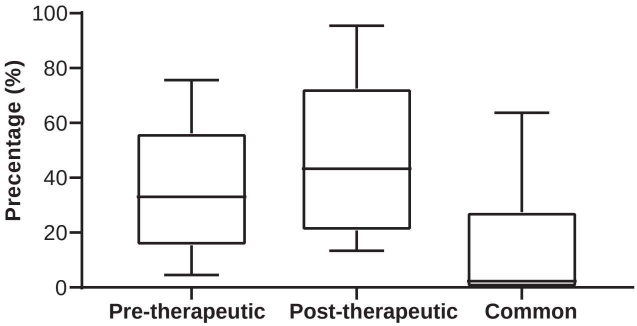
Precentage (%)
100
80
60
40
20
0
Pre-therapeutic
Post-therapeutic
Common

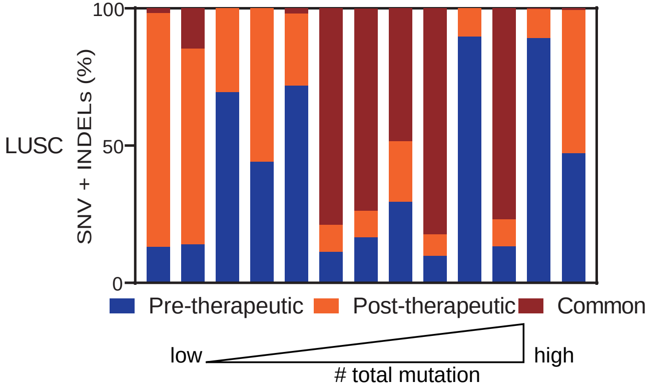
LUSC
SNV + INDELs (%)
100
50
0
Pre-therapeutic
Post-therapeutic
Common
low
high
total mutation

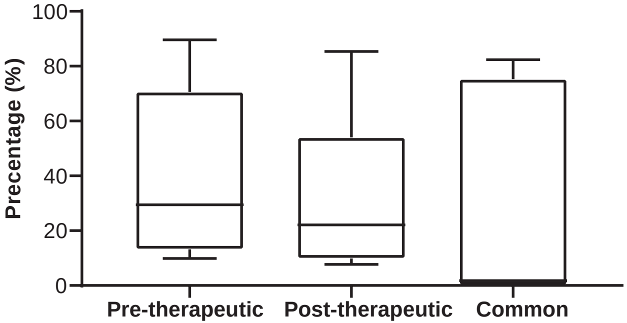
Precentage (%)
100
80
60
40
20
0
Pre-therapeutic
Post-therapeutic
Common

Figure S4

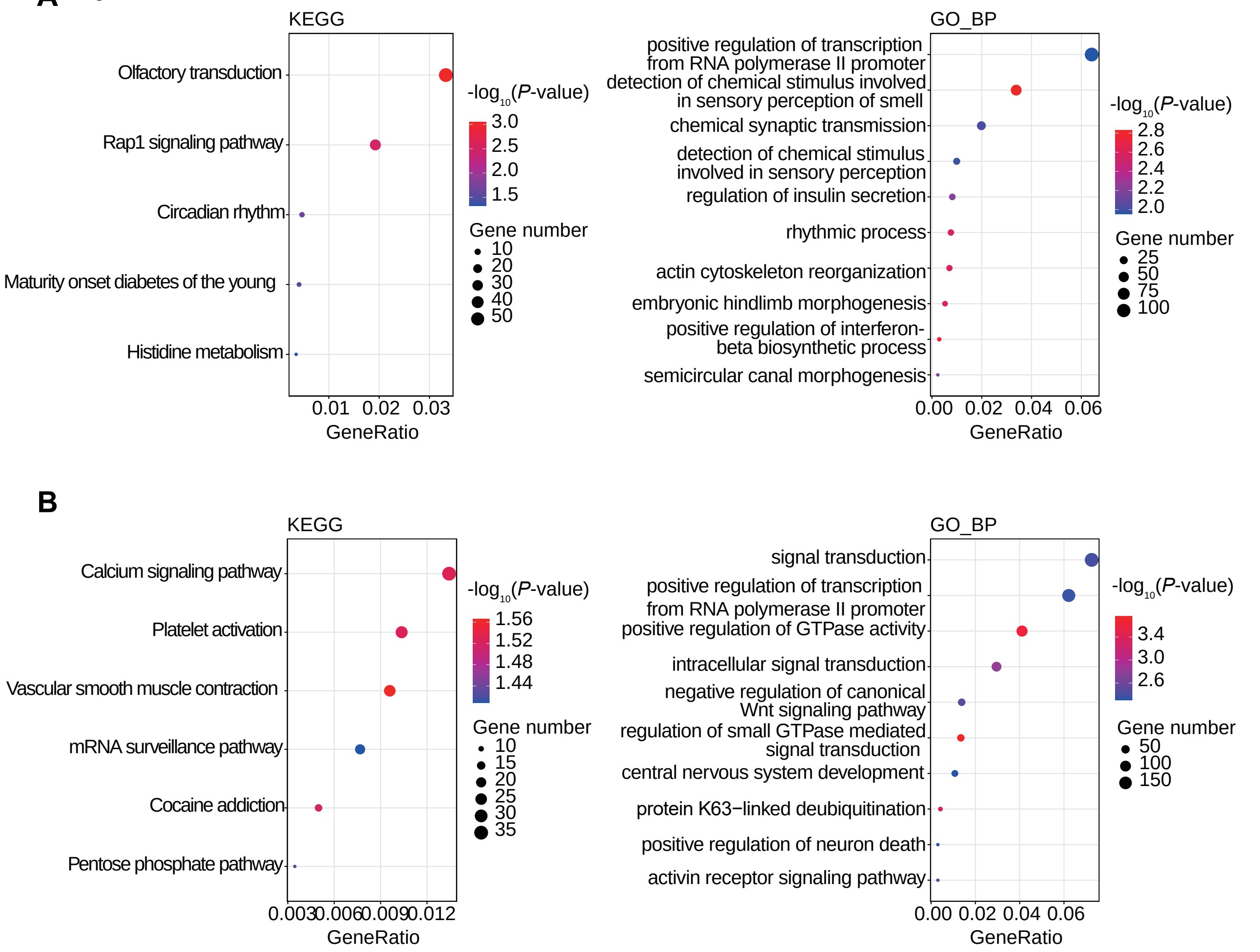
A
KEGG
Olfactory transduction
Rap1 signaling pathway
Circadian rhythm
Maturity onset diabetes of the young
Histidine metabolism
0.01 0.02 0.03
GeneRatio
-log10(P-value)
3.0
2.5
2.0
1.5
Gene number
10
20
30
40
50
GO_BP
positive regulation of transcription from RNA polymerase II promoter
detection of chemical stimulus involved in sensory perception of smell
chemical synaptic transmission
detection of chemical stimulus involved in sensory perception
regulation of insulin secretion
rhythmic process
actin cytoskeleton reorganization
embryonic hindlimb morphogenesis
positive regulation of interferon-beta biosynthetic process
semicircular canal morphogenesis
0.00 0.02 0.04 0.06
GeneRatio
-log10(P-value)
2.8
2.6
2.4
2.2
2.0
Gene number
25
50
75
100
B
KEGG
Calcium signaling pathway
Platelet activation
Vascular smooth muscle contraction
mRNA surveillance pathway
Cocaine addiction
Pentose phosphate pathway
0.003 0.006 0.009 0.012
GeneRatio
-log10(P-value)
1.56
1.52
1.48
1.44
Gene number
10
15
20
25
30
35
GO_BP
signal transduction
positive regulation of transcription from RNA polymerase II promoter
positive regulation of GTPase activity
intracellular signal transduction
negative regulation of canonical Wnt signaling pathway
regulation of small GTPase mediated signal transduction
central nervous system development
protein K63−linked deubiquitination
positive regulation of neuron death
activin receptor signaling pathway
0.00 0.02 0.04 0.06
GeneRatio
-log10(P-value)
3.4
3.0
2.6
Gene number
50
100
150

Figure S5

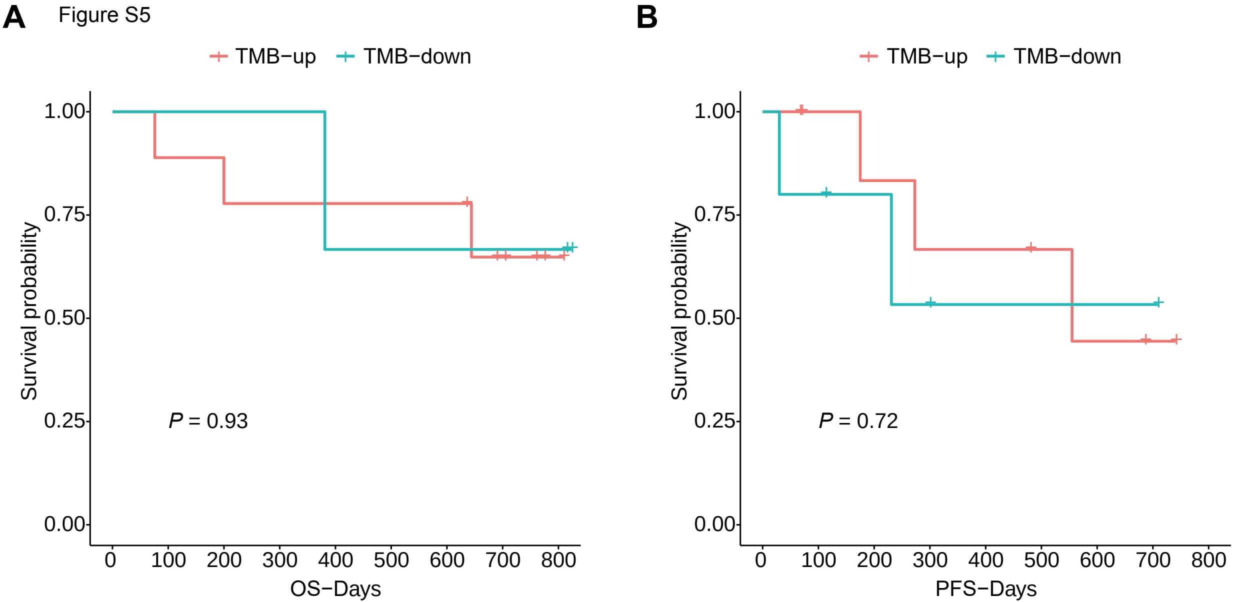
A
TMB−up
TMB−down
Survival probability
1.00
0.75
0.50
0.25
0.00
P = 0.93
0
100
200
300
400
500
600
700
800
OS−Days
B
TMB−up
TMB−down
Survival probability
1.00
0.75
0.50
0.25
0.00
P = 0.72
0
100
200
300
400
500
600
700
800
PFS−Days

Figure S6

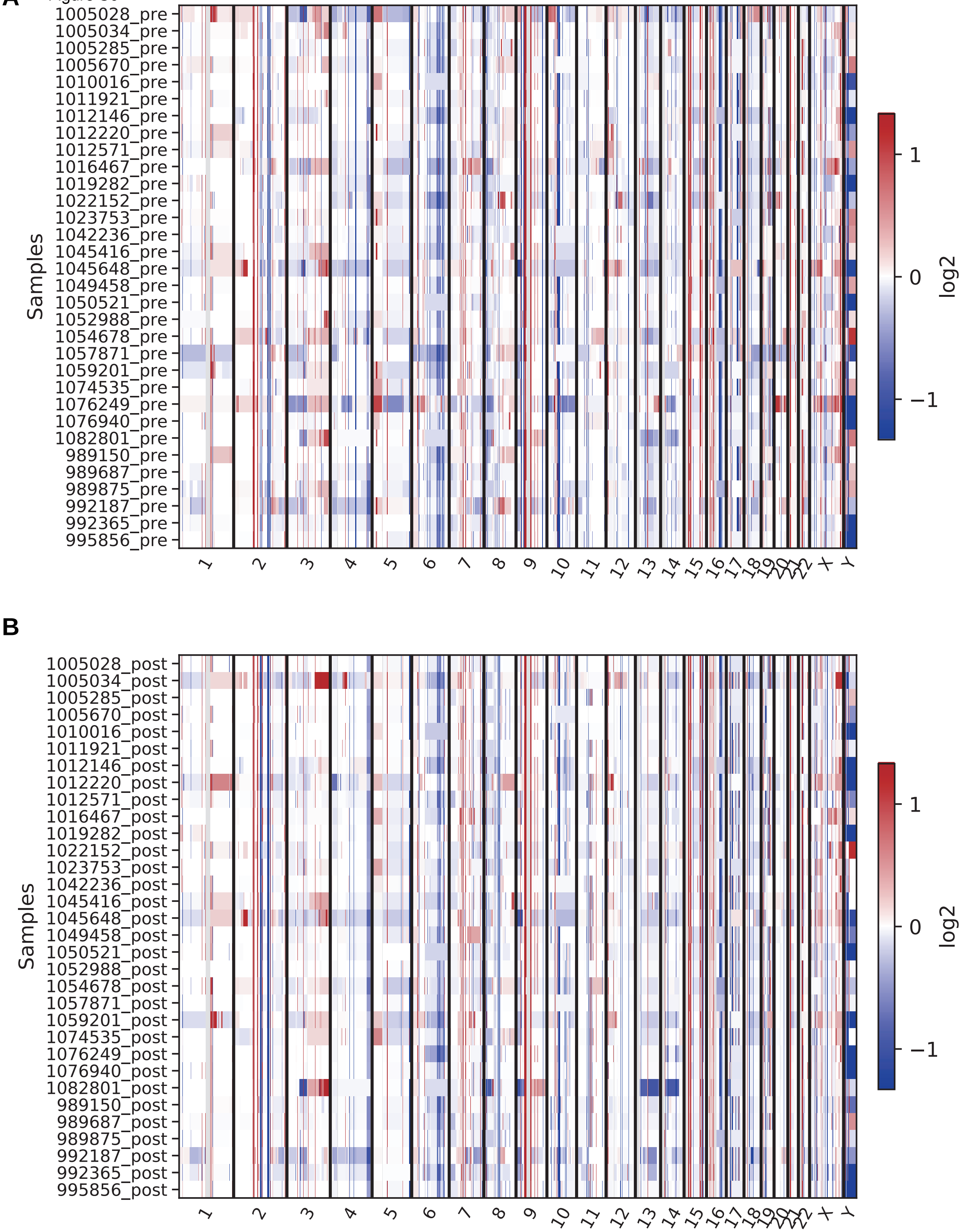
A
Samples
1005028_pre
1005034_pre
1005285_pre
1005670_pre
1010016_pre
1011921_pre
1012146_pre
1012220_pre
1012571_pre
1016467_pre
1019282_pre
1022152_pre
1023753_pre
1042236_pre
1045416_pre
1045648_pre
1049458_pre
1050521_pre
1052988_pre
1054678_pre
1057871_pre
1059201_pre
1074535_pre
1076249_pre
1076940_pre
1082801_pre
989150_pre
989687_pre
989875_pre
992187_pre
992365_pre
995856_pre
1 2 3 4 5 6 7 8 9 10 11 12 13 14 15 16 17 18 19 20 21 22 X Y
1
0
−1
log2
B
Samples
1005028_post
1005034_post
1005285_post
1005670_post
1010016_post
1011921_post
1012146_post
1012220_post
1012571_post
1016467_post
1019282_post
1022152_post
1023753_post
1042236_post
1045416_post
1045648_post
1049458_post
1050521_post
1052988_post
1054678_post
1057871_post
1059201_post
1074535_post
1076249_post
1076940_post
1082801_post
989150_post
989687_post
989875_post
992187_post
992365_post
995856_post
1 2 3 4 5 6 7 8 9 10 11 12 13 14 15 16 17 18 19 20 21 22 X Y
1
0
−1
log2

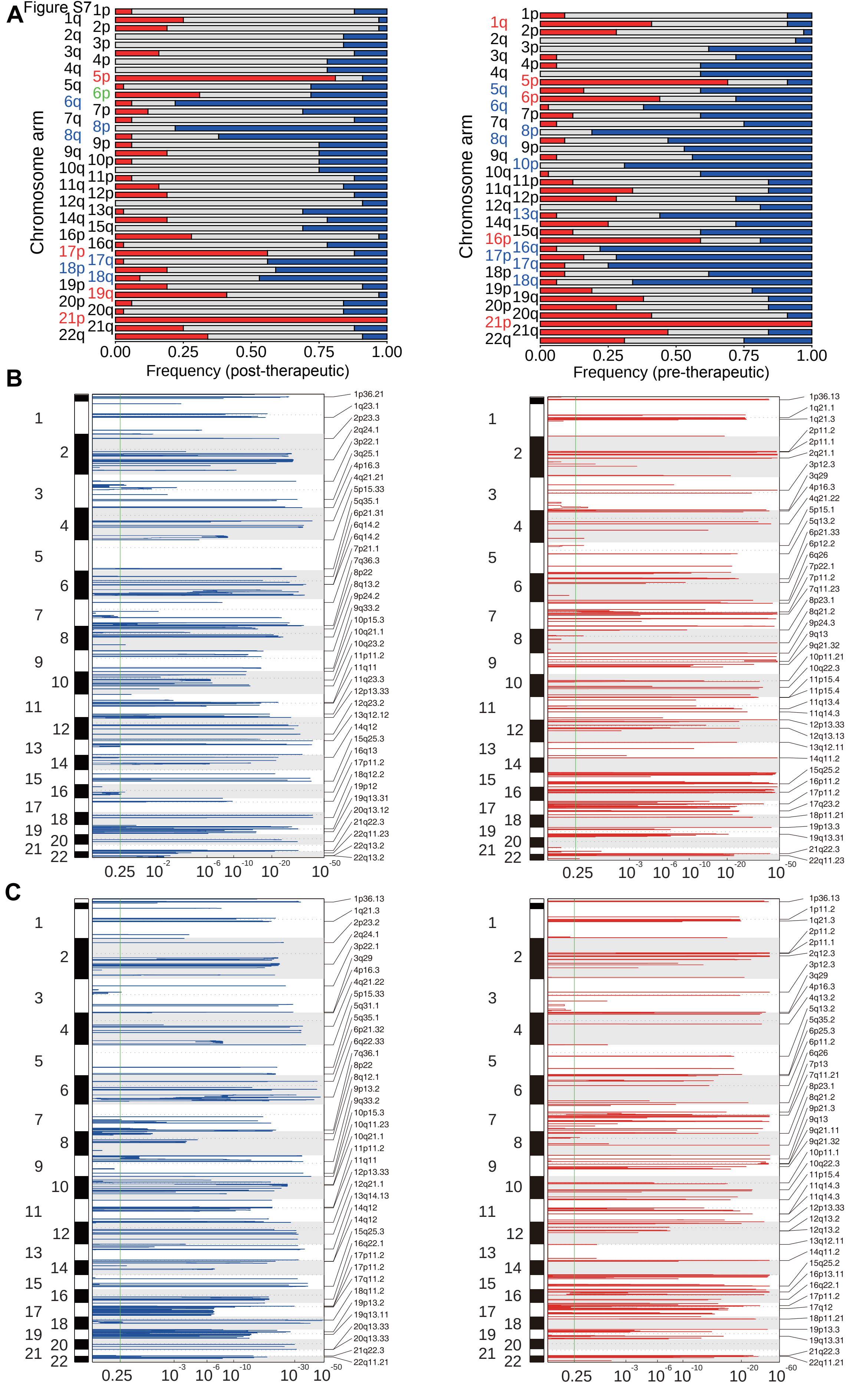
Figure S7
A
Chromosome arm
Frequency (post-therapeutic)
Frequency (pre-therapeutic)
0.00 0.25 0.50 0.75 1.00
B
C
0.25
